# Dual Pili Post-translational Modifications Synergize to Mediate Meningococcal Adherence to Platelet Activating Factor Receptor on Human Airway Cells

**DOI:** 10.1371/journal.ppat.1003377

**Published:** 2013-05-16

**Authors:** Freda E. C. Jen, Matthew J. Warren, Benjamin L. Schulz, Peter M. Power, W. Edward Swords, Jeffery N. Weiser, Michael A. Apicella, Jennifer L. Edwards, Michael P. Jennings

**Affiliations:** 1 Institute for Glycomics, Griffith University, Gold Coast, Queensland, Australia; 2 School of Chemistry Molecular Biosciences, University of Queensland, St Lucia, Brisbane, Queensland, Australia; 3 Molecular Infectious Diseases Group, Department of Paediatrics, Weatherall Institute for Molecular Medicine, University of Oxford, John Radcliffe Hospital, Oxford, United Kingdom; 4 Department of Microbiology, Wake Forest University Health Sciences, Winston-Salem, North Carolina, United States of America; 5 Department of Microbiology, University of Pennsylvania School of Medicine, Philadelphia, Pennsylvania, United States of America; 6 Department of Microbiology, University of Iowa, Iowa City, Iowa, United States of America; 7 The Center for Microbial Pathogenesis, The Research Institute at Nationwide Children's Hospital and the Department of Pediatrics, The Ohio State University, Columbus, Ohio, United States of America; University of Würzburg, Germany

## Abstract

Pili of pathogenic *Neisseria* are major virulence factors associated with adhesion, twitching motility, auto-aggregation, and DNA transformation. Pili of *N. meningitidis* are subject to several different post-translational modifications. Among these pilin modifications, the presence of phosphorylcholine (ChoP) and a glycan on the pilin protein are phase-variable (subject to high frequency, reversible on/off switching of expression). In this study we report the location of two ChoP modifications on the C-terminus of *N. meningitidis* pilin. We show that the surface accessibility of ChoP on pili is affected by phase variable changes to the structure of the pilin-linked glycan. We identify for the first time that the platelet activating factor receptor (PAFr) is a key, early event receptor for meningococcal adherence to human bronchial epithelial cells and tissue, and that synergy between the pilin-linked glycan and ChoP post-translational modifications is required for pili to optimally engage PAFr to mediate adherence to human airway cells.

## Author Summary

*Neisseria meningitidis* is an important human pathogen that can cause rapidly progressing, life threatening meningitis and sepsis in humans. There is no fully protective vaccine against this pathogen in current use and the key processes that dictate the transition from harmless carriage of the bacterium in the airway (the case for the vast majority of colonised hosts) to invasive disease are largely undefined. A key missing link in this organism's interaction with the human host is the identity of the receptor that is the first point of contact for the organism within the airway. In this study, we report that the receptor for this important human pathogen on airway epithelial cells is the platelet activating factor receptor (PAFr), an immunomodulatory molecule shown by others to play a role in promoting bacterial sepsis. We also show that two post-translational modifications, glycosylation and phosphorylcholine, are subject to phase-variation (high frequency, reversible switching of gene expression). They are closely associated on adjacent pilin subunits, and synergy between both are required for the efficient engagement with the PAFr. These data define a new role for these post-translational modifications in meningococcal adherence and also provide an insight into the selective pressures that underlie their phase variable expression.

## Introduction

Type IV fimbriae, or pili, are long filamentous structures that extend from the bacterial surface and primarily consist of the monomer pilin protein [1]. Pili are shown to play a major role in promoting colonization of the mucosal epithelium by several human pathogens and, thus, are generally considered to be major virulence determinants for bacterial pathogens. Roles attributed to the Type IV pili expressed by the pathogenic *Neisseria* (*N. gonorrhoeae* and *N. meningitidis*) include: adhesion, cytotoxicity, twitching motility, auto-aggregation, and DNA transformation [2] [3] [4]; [5]. Pili of the pathogenic *Neisseria* are post-translationally modified. These post-translational modifications (PTMs) include: a glycan [6] [7] [8], phosphorylcholine (ChoP) [9], and/or a phosphoglycerol [10]. Roles for these PTMs in the pathogenesis of *N. gonorrhoeae* and *N. meningitidis* are proposed. For example, during *N. gonorrhoeae* challenge of primary human cervical epithelial cells, the pilin glycan mediates the activation state of complement receptor 3 (CR3); this, in turn, modulates gonococcal adherence, invasion, and ultimately their intra-epithelial cell survival [11]. Additionally, the pili-linked phosphoglycerol is proposed to trigger *N. meningitidis* dissemination [10]. The role of the ChoP PTM has not been previously determined and is the subject of this study.

In *N. meningitidis* strain C311#3, pilin is glycosylated at Ser63 with the trisaccharide, Gal(β1-4)Gal(α1-3)2,4-diacetimido-2,4,6-trideoxyhexose (DATDH) [6]. We previously describe a series of gene products (PglB, PglC, PglD) involved in the biosynthesis of DATDH [12]. Once formed, DATDH serves as the base for the stepwise development of the trisaccharide, in which PglA transfers the first galactose (Gal(α1-3)) to the DATDH [13] that is followed by the PglE-mediated addition of a second (terminal) galactose (Gal(β1-4)) to form the mature trisaccharide [12]. Not all strains of *N. meningitidis* make this same glycan. *Neisseria* are capable of the biosynthesis of a wide range of glycans that can be transferred to protein targets, which is dependent upon the absence, or presence, of various glycosyltransferases in any particular strain (*e. g.*, PglG, PglH, PglE); and also because many of these glycosyltransferase genes (*pglA*, *E*, *I*, *G*, and *H*) are subject to phase variation (random ON/OFF switching of expression) [13] [14] [8] [15].

ChoP is another phase variable PTM made to the pilin of the pathogenic *Neisseria* [16] [9] [17]. Several microorganisms of the human respiratory tract express ChoP on their surface where it serves as a ligand to mediate an association with a host cell [18] [19] [20] [21] [22]. In both non-typeable *Haemophilus influenzae* (NTHi) and the commensal *Neisseria*, ChoP is important in adherence via the platelet activating factor receptor (PAFr) as well as in the signalling cascades that ultimately result in invasion of some epithelial cells [23], [24]. Alternatively, ChoP can also serve as a target for bacterial killing by C-Reactive Protein (CRP). For example, CRP binds to ChoP present on *Streptococcus pneumoniae* [25], *H. influenzae* [22], commensal *Neisseria* [24], and *N. meningitidis* [26]. Although not yet show for *Neisseria spp.*, within the nasopharynx, CRP binding to ChoP can decrease bacterial adherence to the PAFr [27]. Within the blood, opsonization with CRP can activate complement by the classical pathway, resulting in a bactericidal effect [22]. Therefore, the phase variable expression of ChoP is a seemingly critical mechanism in the colonization and pathogenesis of bacteria commonly found within the human airway [9].

In this study, we hypothesized that the phase variable exposure of ChoP on pili of *N. meningitidis* may be of functional importance in aiding colonization and in immune evasion. To test this hypothesis, we first set out to define the surface accessibility of ChoP and the precise location of the ChoP PTM on pilin. We then examined the role of the pilin-linked glycan and ChoP as contributors to meningococcal adherence to the PAFr on a PAFr-expressing cell line and, of substantive importance to human disease, to the PAFr present on human bronchial epithelial cells and on human bronchial tissue.

## Results

### ChoP is a common modification on *N. meningitidis* pili

**Figure 1:**
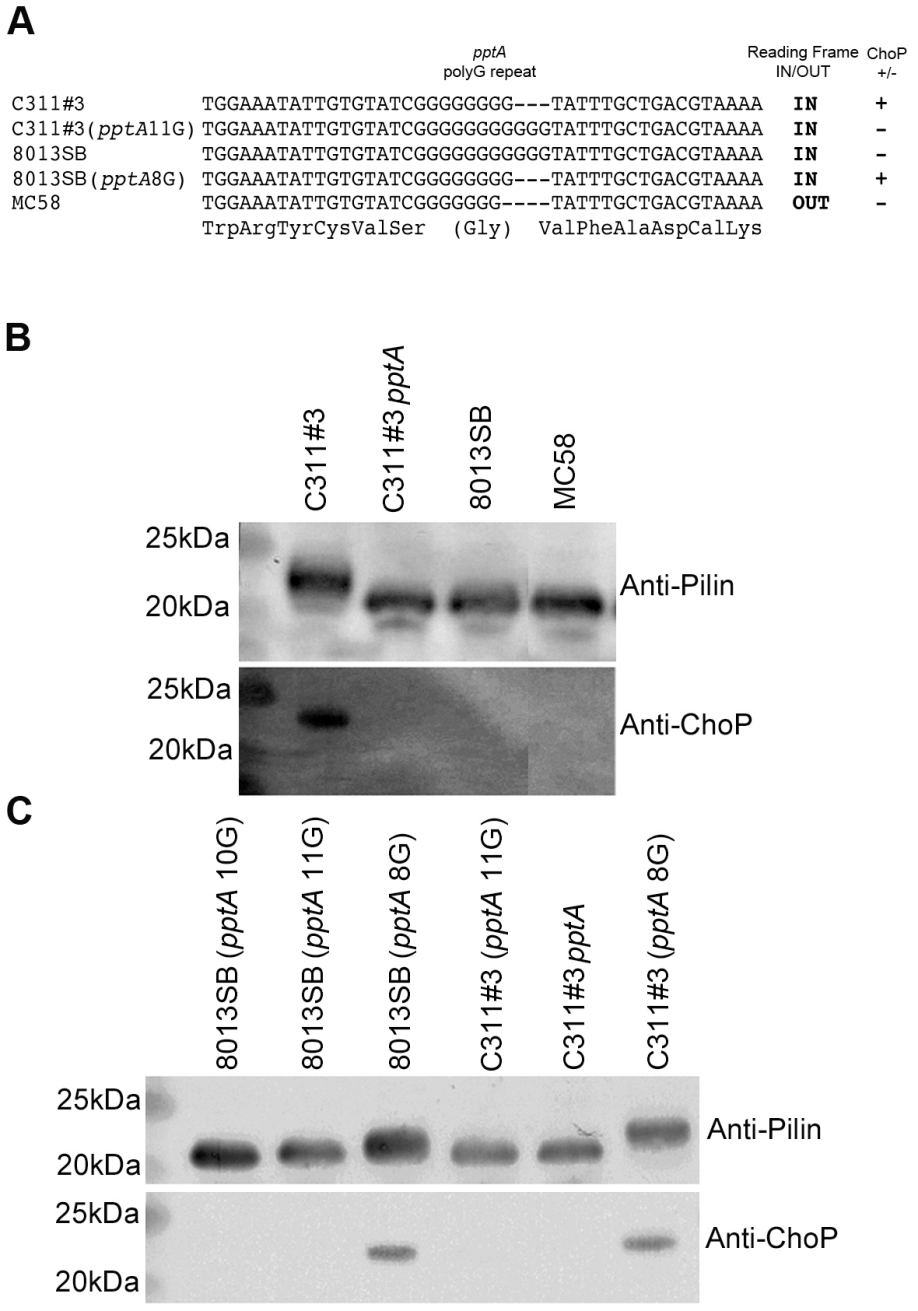
Presence of ChoP on pili in pathogenic *N. meningitidis* and its mediation by phase variation of the transferase, PptA. A) Sequencing of the polymeric (G) tract region of various *N. meningitidis* strains and mutants with altered tract lengths. ON and OFF, respectively, indicate whether the gene is in-frame or out of frame for expression of a functional protein (translation of in-frame sequence shown under nucleotide sequence), ChoP PTM on pili from these strains is indicated as being present (+) or absent (−). B) Western blotting demonstrates the presence of ChoP on *N. meningitidis* pili and (C) the effect of changes in tract length on PptA function. Collectively, these data show that pili of *N. meningitidis* can only be modified with ChoP when the *pptA* polymeric repeat tract contains 8 glycines.

We screened a representative collection of 32 *N. meningitidis* strains that were chosen for temporal, geographic and genetic diversity (from a WHO collection plus our own clinically isolated bacteria; [28]). All of the strains tested contained the gene, *pptA* (NMB0415), encoding the transferase responsible for the addition of ChoP to pili (for pathway see [29]); thus, every strain examined had the potential for pili to be post-translationally modified by ChoP [17]. However, the gene encoding PptA contains a homopolymeric tract of guanosine (G) residues within its coding region (encoding poly-glycine, see Fig. 1A), which are hypermutagenic, leading to frame-shift mutations and, in turn, the reversible ON/OFF switching of gene expression (phase variation) and, thus, of ChoP addition to pili [17]. Pili may also phase vary ON and OFF at a high frequency, and although essential for adherence in colonisation of the host, may phase vary to OFF during *in vitro* culture (reviewed by [30]). Analysis of the pili expressing strains within the WHO and clinical isolate collection revealed that the majority had *pptA* genes possessing 8G residues, which is in-frame for expression of this gene (Fig. 1A). Of those strains that did not express ChoP on their pili, the majority possessed homopolymeric tract numbers (7G, 9G, 10G) that were out-of-frame for expression (Fig. 1A, Tbl. S3). Strains containing an 11G tract in *pptA* had no detectable expression of ChoP on their pili (see Fig. 1B for example). This was surprising because 11G is in-frame for PptA expression. Within the PptA polypeptide, an 11G homopolymeric region should result in the generation of a full-length protein with only one additional glycine, compared to those strains harbouring an 8G *pptA* homopolymeric region (Fig. 1A). To determine whether the extra glycine, resulting from the difference in the 8G *vs.* 11G tract length within the *ppt*A homopolymeric region, was responsible for the observed loss of PptA protein function; the number of Gs comprising the *pptA* homopolymeric region of strains C311#3 (*pptA*8G, ChoP+; our model strain [17]) and 8013SB (*pptA*11G, ChoP−; another model strain [10]) were genetically altered such that C311#3 now contained 11Gs (*i. e.*, C311#3*pptA*11G) and 8013SB now contained 8Gs (*i. e.*, 8013SB*pptA*8G). We found that, although both 8G and 11G versions of *pptA* are in-frame for PptA expression, ChoP was only detectable on pili when *pptA* contained 8Gs (Fig. 1C). This demonstrated that the 8013SB *pptA* gene is functional when modified to contain one less glycine, and, therefore, that the number of glycines expressed is critical for PptA function. Colony immunoblot screening of approximately 80,000 wildtype (WT) 8013*pptA*11G colonies failed to yield any phase variants that had reverted to a ChoP positive phenotype. This suggested that a phase variation event that would allow ChoP expression to switch back ON (*pptA*11G to *pptA*8G) is rare. Such an event would require 3 independent mutational events; 11G to 10G, 10G to 9G, and then 9G to 8G; to achieve expression of an active PptA. In contrast, strains with the out-of-frame forms, *pptA*7G or *pptA*9G, require only a single mutational event to switch to the in-frame, active, *pptA*8G form. Thereby, the latter *pptA*7G or *pptA*9G out-of-frame form is easily reversible, with ON/OFF switching occurring at the approximate rate of 1 in 100 colonies. Our analysis of “G” repeat numbers within *pptA* indicated that the *pptA*11G strains; which express the inactive PptAGly^+1^ form of the enzyme and, therefore, do not express ChoP on their pili; are uncommon and represent only 6% of the clinical isolates surveyed (See Table S3). This, thereby, suggests that the ChoP PTM is an important aspect of pili function.

### Accessibility to pilin-linked ChoP is mediated by ChoP-independent changes to pili

**Figure 2:**
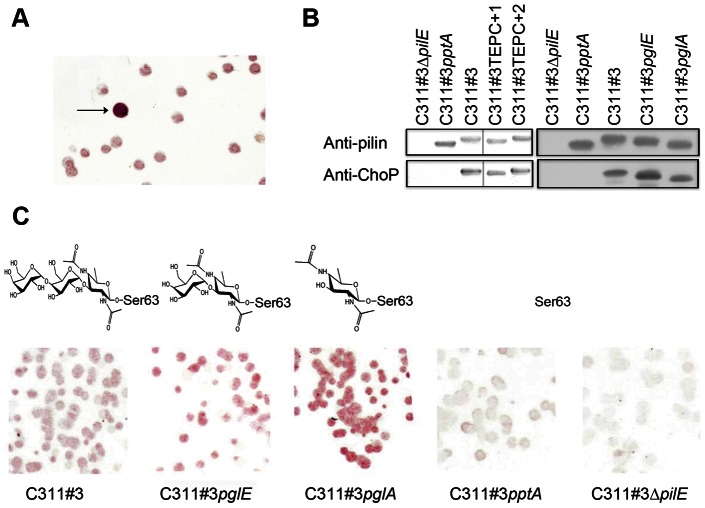
Analysis of denatured pilin from *N. meningitidis* C311#3 and its TEPC-15 variants. (A) Colony immunoblotting of *N. meningitidis* C311#3 with anti-ChoP (TEPC-15) antibody revealed a colony with increased immunoreactivity and is indicated by the arrow. (B) Pilin was isolated from each of the mutants noted and examined under denaturing conditions by western blotting. No difference in TEPC-15 binding was observed between C311#3 WT, glycosylation mutants (C311#3*pglE* and C311#3*pglA*), or TEPC variants (C311#3TEPC+1 and +2). TEPC-15 did not bind to meningococci in which pili were not expressed (C311#3Δ*pilE*) or in which the ability of ChoP PTM was lost (C311#3*pptA*) (C). Colony immunoblotting of C311#3 glycosylation mutants shows that TEPC-15 reactivity increases as the pilin linked glycan decreases in size.

Using the ChoP-specific, monoclonal antibody, TEPC-15, we assessed the surface accessibility of ChoP on native pili. ChoP was detectable on the majority of *N. meningitidis* strain C311#3 with some colonies exhibiting a much higher level of TEPC-15 binding (Fig. 2A). This suggested that either more ChoP was present on the surface of some bacterial cells or that the altered presentation of ChoP on the surface of some bacteria resulted in an increase in antibody accessibility. Therefore, we isolated these highly TEPC-15-reactive colonies to determine whether the increase in TEPC-15 reactivity was attributable to an increase in ChoP on each pilin subunit. Western Blot analysis of denatured pili revealed equivalent TEPC-15 reactivity among the various pili tested (Fig. 2B). This indicated that the amount of ChoP present on pili was not responsible for the increase in TEPC-15 reactivity observed by Colony Blot analysis. Rather, these data suggested that high antibody reactivity likely resulted from differences in ChoP accessibility to TEPC-15 in the context of the properly folded, native pili polymer. Such differences in ChoP accessibility could have resulted from variation in the pilin primary amino acid sequence and/or from variation in other, additional, pilin PTMs reported for meningococci. To determine the mechanism(s) of altered ChoP accessibility, both of these possibilities were examined.

### Antibody accessibility to ChoP results from changes in the pilin primary sequence and by phase variation of the pilin-linked glycan

The gene encoding pilin, *pilE*, can alter its sequence at high frequency by recombination with silent pilin gene copies, called *pilS* [30]. To determine whether changes in the amino acid sequence of PilE contributed to increased TEPC-15 reactivity, the *pilE* sequences from three (C311#3TEPC+1, C311#3TEPC+2, and C311#3TEPC+3) high TEPC-15 reactive colonies were examined. Sequencing of *pilE* revealed that nucleotide variation, consistent with amino acid alterations, in *pilE* had occurred in two variants, C311#3TEPC+1 and C311#3TEPC+2 (Fig. S1). No changes had occurred in the expression of phase variable *pgl* genes in these two strains and pili-linked trisaccharide was evident in Western blot analysis using anti-trisaccharide sera (result not shown). Upon mapping these variations onto the described structural model of the pilus fiber [31], we found that, within the assembled pilus fibre, these changes occurred near the site of pilin glycosylation. Conversely, no amino acid changes were observed for C311#3TEPC+3, indicating that an additional mechanism(s) of altered accessibility to ChoP existed. Altered accessibility to ChoP could result from variations in other PTMs, *e. g.*, phase variation of the glycosyltransferases involved in the biosynthesis and, thus, the expression, of the pilin-linked glycan. To determine if the phase variable expression of the pilin-linked glycan also contributed to the accessibility of ChoP on the pilus fiber, we sequenced the repeat tract regions that control phase variation of the pilin glycosylation genes, *pglA* [13] and *pglE* [32]. The repeat region of *pglE* from strain C311#3TEPC+3 had indeed lost a repeat unit leading to a frame-shift mutation and an inactive PglE. As phase variation of *pglE* to an “off” state results in the loss of the terminal galactose from the pilin-linked trisaccharide (Fig. 2C), these data provided the first evidence that alterations to the trisaccharide structure may influence the accessibility of ChoP on pili.

Using defined pilin glycosylation mutants, we then analysed native pili for the effect of glycan truncation on ChoP exposure by colony immunolabeling. *N. meningitidis* strains tested included C311#3 [6] and its previously described mutants, C311#3*pglE* [12] and C311#3*pglA* [13]. The C311#3*pptA* mutant (lacking ChoP; [17]) and C311#3Δ*pilE* mutant (lacking pili [33]) were included as negative controls. TEPC-15 bound only weakly to C311#3 WT colonies and did not bind to the negative control strains, C311#3*pptA* and C311#3Δ*pilE* (Fig. 2C). The remaining strains exhibited an increase in the level of TEPC-15 bound, and this was inversely proportional to the length of the pilin-linked glycan expressed by each mutant strain. The C311#3*pglE* mutant (possessing a pili-linked disaccharide) displayed a slight increase in TEPC-15 binding when compared to the WT. There was a further increase in TEPC-15 reactivity for the C311#3*pglA* mutant (glycosylated with a monosaccharide) [13].

To confirm that the observed hierarchy of TEPC-15 binding to ChoP resulted from interactions present in the pili polymer, pilin was isolated from each of the mutants and examined under denaturing conditions by western blotting. No difference in TEPC-15 binding was observed between C311#3 WT, C311#3*pglE*, and C311#3*pglA* mutants (Fig. 2B). However, we observed a hierarchy in TEPC-15 binding to ChoP under native conditions (Fig. 2C). We propose that these two PTM structures are closely associated and that masking of ChoP by the pili-linked glycan alters the accessibility of ChoP in the native pilus fibre.

### Post-translational modification of pilin with ChoP occurs at the C-terminus

**Figure 3:**
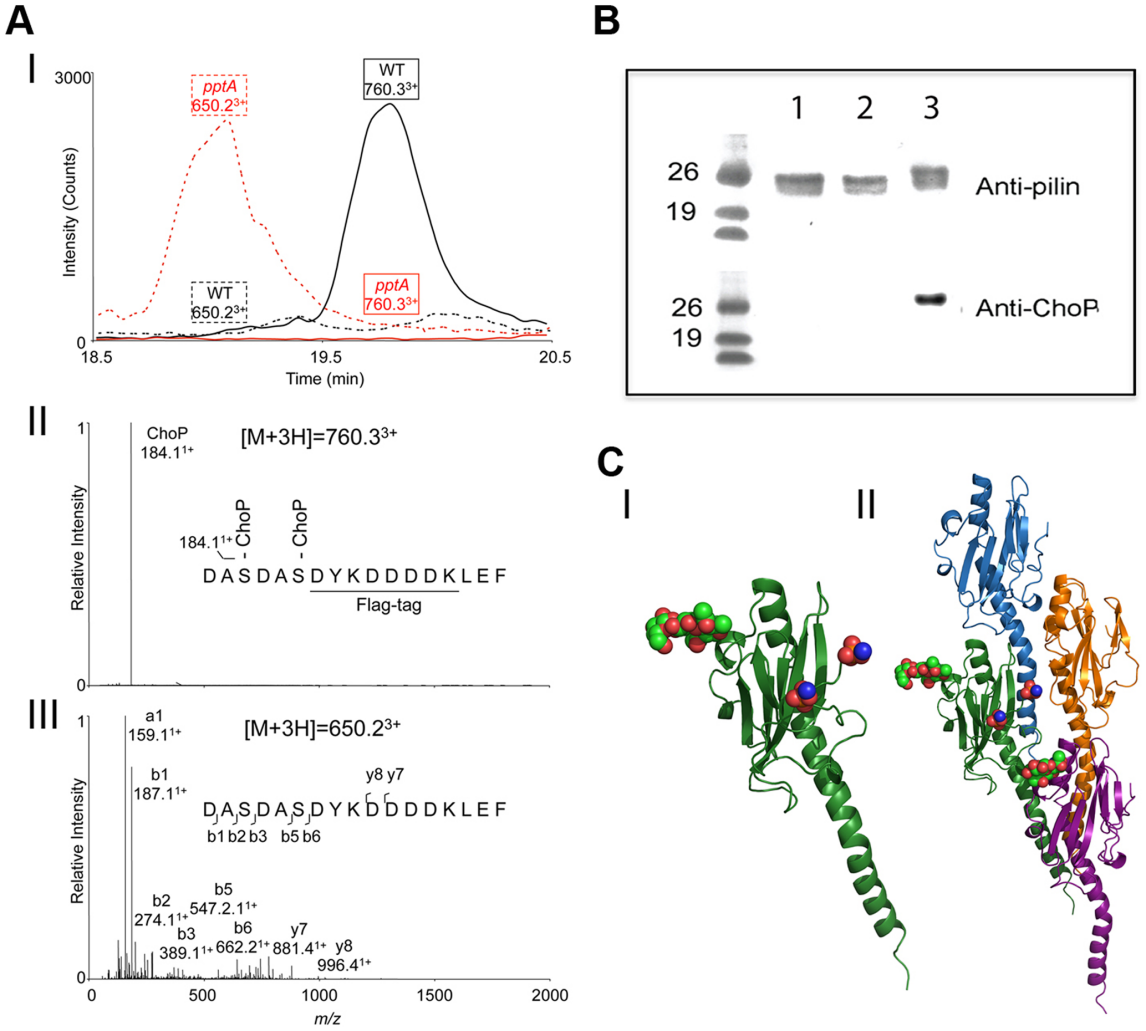
Analysis of site of ChoP addition in *N. meningitidis* C311#3. (A) LC-MS/MS of trypsin-digested purified pilin showed that two ChoP residues are attached to Ser157 and Ser160 in WT C311#3. (I) Extracted ion chromatograms of ChoP-modified (m/z of 760.3) and unmodified pilin peptides (m/z of 650.2) in C311#3 WT and C311#3*pptA* purified pilin. (II) MS/MS of ChoP modified pilin peptides of C311#3 WT. The MS/MS of [M+3H]^3+^ signal at m/z of 760.3 suggested this peptide is modified with two ChoP, which has an ion mass of 184.1^+^ Da. (III) LC-ESI/MS analysis of trypsin-digested pilin from C311#3*pptA lacked* ChoP on pilin as indicated by the unmodified ^155^DASDASDYKDDDDKLEF^170^ peptide [M+3H]3+ ion at m/z of 650.2 (neutral mass of 1947.6 Da) and the absence of [M+3H]3+ ion at m/z 760.3. (B) Western Blot analysis of pilin from *N. meningitidis* C311#3 S157A/S160A mutant (no FLAG-tagged) demonstrates that ChoP is only present on Ser 157 and Ser 160. Lane 1 – C311#3S157A/S160A, lane 2 – C311#3*pptA* pilin, lane 3 – WT pilin. (C) Modelling of the pili-linked glycan (Ser63; red/green spheres) and ChoP (red/blue spheres) on the C-terminus of the pilin subunit (I) demonstrates their juxtaposition when adjacent pilin subunits are stack in the pili polymer (II).

Our data indicated that the ChoP and glycan structures are likely juxtaposed on the mature pilus fibre. Within the gonococcal pilus structure [34], Ser 34, 45, 68, 69, 70, 157, and 160 are all located near the site of glycan addition. In that the site of ChoP addition to pilin is unknown, we made Ser/Ala conversion mutants in strain C311#3 by site-directed mutagenesis. Each of these mutants was then analysed by western blotting for the presence of ChoP. TEPC-15 antibody still recognized each isolated mutant pilin form, indicating ChoP was still present. Hence, we then generated and purified (C311#3 WT and C311#3*pptA* mutant) pilin fused to a FLAG-tag (Fig. S2) to enable identification of the ChoP PTM site by LC-MS/MS (Fig. 3A). LC-ESI/MS analysis of trypsin-digested, FLAG-tagged pilin revealed that no molecular ion was present at the calculated mass for the pilin C-terminal trypsin-digested peptide, ^155^DASDASDYKDDDDKLEF^170^ (1947.8 Da). Instead, an unassigned LC-ESI/MS [M+3H]^3+^ signal at m/z of 760.3 was observed for C311#3WT pilin; whereas, 650.2 m/z was observed for the pilin from the C311#3*pptA* mutant strain (Fig. 3A (panel I)). The neutral mass of [M+3H]^3+^ at m/z of 760.3 is 2277.9 Da. The difference between the observed mass (2277.9) and the predicted mass (1947.8) is 330.1 Da, or the equivalent of two molecules of 165 Da. When the covalent bond between ChoP and a serine residue breaks, the mass of ChoP then becomes 184.1^+^ Da (162Da+18Da(H_2_O)+1Da; ChoP tends to exist as positive ion) (Fig. 3A, panel II). The LC-ESI/MS result of trypsin-digested pilin from C311#3*pptA* lacking ChoP showed an unmodified ^155^DASDASDYKDDDDKLEF^170^ peptide [M+3H]3+ ion at m/z of 650.2 (neutral mass of 1947.6 Da) and no observation of an [M+3H]3+ ion at m/z 760.3. In Fig. 3A (panel III), the MS/MS data confirmed the sequence of a ChoP unmodified ^155^DASDASDYKDDDKLEF^170^ peptide. There was a two ChoP mass difference between the modified (760.33^+^ m/z) and unmodified (650.23^+^ m/z) peptides, which indicates that ChoP is attached to both Ser 157 and Ser 160.

### The C-terminus of pilin serves as the only site for ChoP modification

LC-MS/MS analyses indicated that a ChoP is attached to both Ser157 and Ser160. However, as pilin containing a FLAG-tag at its C-terminus (*i. e.*, after Ser160) was used for these experiments, we wanted to confirm that data obtained were attributable to pilin (not the FLAG motif), as well as to ensure that Ser157 and Ser160 were the only sites of ChoP modification. To this end, we generated a S157A/S160A mutant strain of C311#3, C311#3S157A/S160A, by site-directed mutagenesis. Pilin of C311#3S157A/S160A was then isolated and analysed by probing Western Blots with anti-pilin or anti-ChoP (TEPC-15) antibody. Although membranes probed with the anti-pilin serum showed the presence of pilin in each sample, ChoP was not detectable on strain C311#3S157A/S160A (Fig. 3B). These data confirmed that ChoP modifications occurred only on Ser 157 and Ser 160 of the pilin protein.

### Molecular modelling indicates ChoP modifications on the pilin C-terminus are masked by the pili-linked glycan

We have proposed that masking of ChoP by the pili-linked glycan alters the accessibility of ChoP (to TEPC-15) within the pilus fibre. Having defined the sites for pilin ChoP modification, we next wanted to investigate how ChoP and the pilin-linked glycan may be potentially associated in the context of the pilus polymer. Although the structure of *N. meningitidis* pilus is not available, it has been modelled based on the published *N. gonorrhoeae* pilus crystal structure [34]. Based on this same model, and by using the molecular modelling program Insight II (Accelrys), we altered the C-terminal region of *N. gonorrhoeae* pilin to possess a RDASDAS motif (consistent with C311#3 pilin) with two serine-linked ChoP modifications. Additionally, a trisaccharide structure, also consistent with C311#3, was added to Ser 63. The structure was calculated in the minimal energy condition. As shown in Fig. 3C (panel I) and in supporting information (Movie S1), a long distance exists between the glycan present on Ser 63 and the ChoP moieties present on the C-terminus. Further, the glycan and ChoP modifications are not on the same side of pilin protein. However, when multiple pilin subunits come together to form a pilus fiber, the pilin glycan is proximal to the ChoP in the adjacent pilin subunit (Fig. 3C, panel II and supporting information Movie S2). Therefore, the observed altered surface accessibility to ChoP on the C-terminus of any one pilin monomer most likely results from variation in the glycan on the adjacent pilin subunit.

### ChoP and glycan moieties contribute to a PAFr-Pilus interaction *in vitro* and on human bronchial epithelial cells

The above data support the hypothesis that the ChoP and glycan PTMs are closely associated in the native pilus fibre. Thus, investigations of the biological role of ChoP must take into account, phase variable, glycan structural variations. To determine the biological significance of the ChoP PTM of pili, we investigated the interaction of meningococci with the PAFr on human bronchial epithelial cells, as there are ample data to indicate that the PAFr serves as a docking site for molecules on which ChoP is exposed [23]; [24].

**Figure 4:**
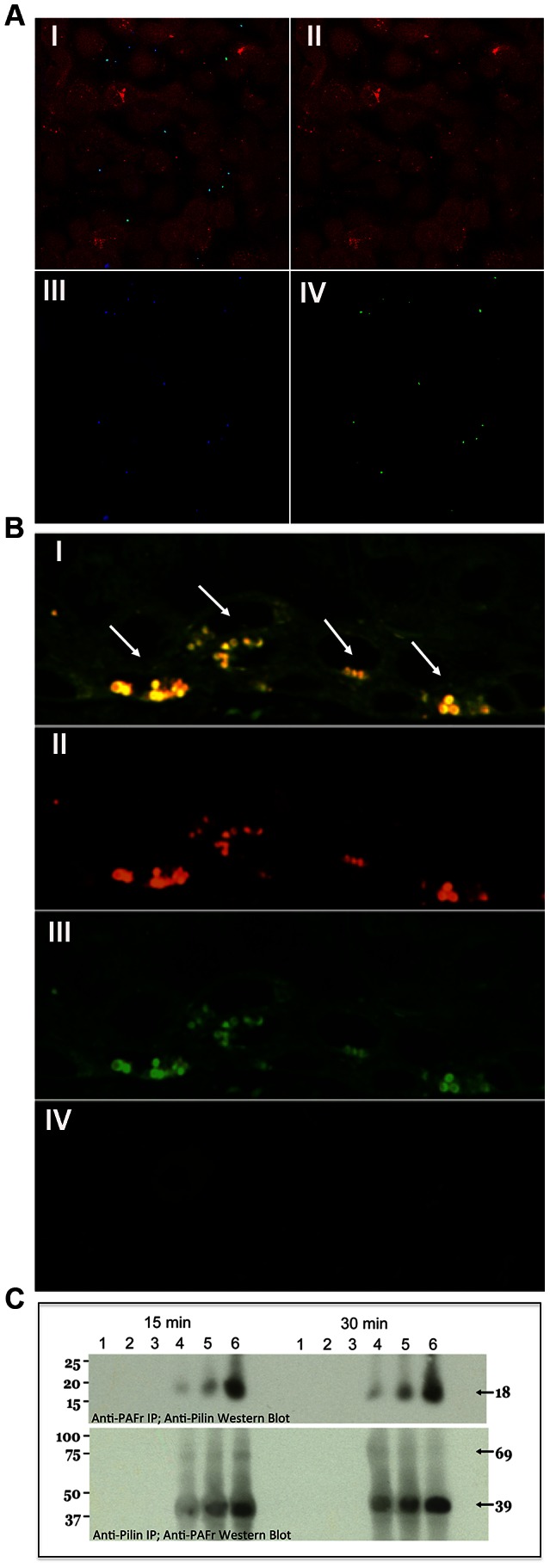
*N. meningitidis* C311#3 pilin-linked ChoP mediates adherence to the PAFr on human airway epithelial cells. (A) Confocal microscopy demonstrates that nearly 100% of *N. meningitidis* C311#3*gfp* co-localize with the PAFr on 16HBE14 cells at 15 minutes post-infection. Panel (I) shows the merged image of red, blue, and green channels. Panel: (II) Red channel - Cell Tracker-stained 16HBE14 cells, (III) Blue channel - PAFr, (anti-PAF rabbit sera+Alexa 647 goat anti-rabbit), (IV) Green channel - GFP-expressing C311#3. (B) Co-localization of C311#3 WT (red) with the PAFr (green) is readily visible by confocal microscopy as a yellow fluorescence (denoted by arrows) following a 30 minute infection of human bronchial tissue. Panel (I) shows the merged image of the red and green channels. Panel: (II) Red channel - *N. meningitidis* immunolabeled with antibody pS-20 and a rhodamine-conjugated secondary antibody, (III) Green channel - the PAFr immunolabeled with primary antibody H-300 and a FITC-conjugated secondary antibody (IV) Control in which both primary antibodies have been omitted. (C) IP using anti-pilin (top panel) or anti-PAFr (bottom panel) antibodies demonstrates a direct interaction occurs between the PAFr and pilin at 15 and 30 minutes post-challenge of 16HBE14 cells. Lanes: (1) the primary antibody was omitted from the capture step, (2) the secondary, agarose-conjugated, antibody was omitted from the assay, (3) uninfected 16HBE14 cells, infections were performed using (4) strain C31126A (trisaccharide, no ChoP), (5) strain C311*pglA* (disaccharide, ChoP+), and (6) WT C311#3 (trisaccharide, ChoP+). These data show that both ChoP and the pilin-linked glycan are required for efficient binding to the PAFr on human bronchial cells.

Confocal microscopy revealed that, after 15 minutes incubation, 100% of cell associated *N. meningitidis* C311#3 were co-localized with the PAFr on 16HBE14 human airway epithelial cells (Fig. 4A). Similar data were observed upon the analysis of human bronchial tissue that had been challenged for 30 min with WT meningococci (Fig. 4B). Co-localization was not observed in tissue sections in which the primary anti-meningococcal and -PAFr antibodies were omitted during processing (negative control; Fig. 4B). These data were supported by further quantitative investigations to determine whether a pili-linked ChoP-PAFr interaction accounted for the co-localisation of *N. meningitidis* with the PAFr that we observed by confocal microscopy. To this end, PAFr and pilus immune complexes were captured from uninfected 16HBE14 cells by immunoprecipitation (IP), as well as from 16HBE14 cells that were challenged for 15 min (Fig. S3) or 30 min with *N. meningitidis* strains C311#3 WT, C311#326A (trisaccharide, no ChoP), or C311#3*pglA* (disaccharide, ChoP+) (Fig. 4C). Western Blots in which pilus-associated complexes were probed with an anti-PAFr antibody (C-20), revealed a prominent band at approximately 39 kDa, as well as a fainter band of approximately 69 kDa, consistent with the PAFr (Fig. 4C). Reciprocal experiments, in which anti-PAFr IPs were probed for the presence of pilus, demonstrated similar results in that an approximate 18 kDa band, suggestive of pilus, was readily evident (Fig. 4C and S3).

**Figure 5:**
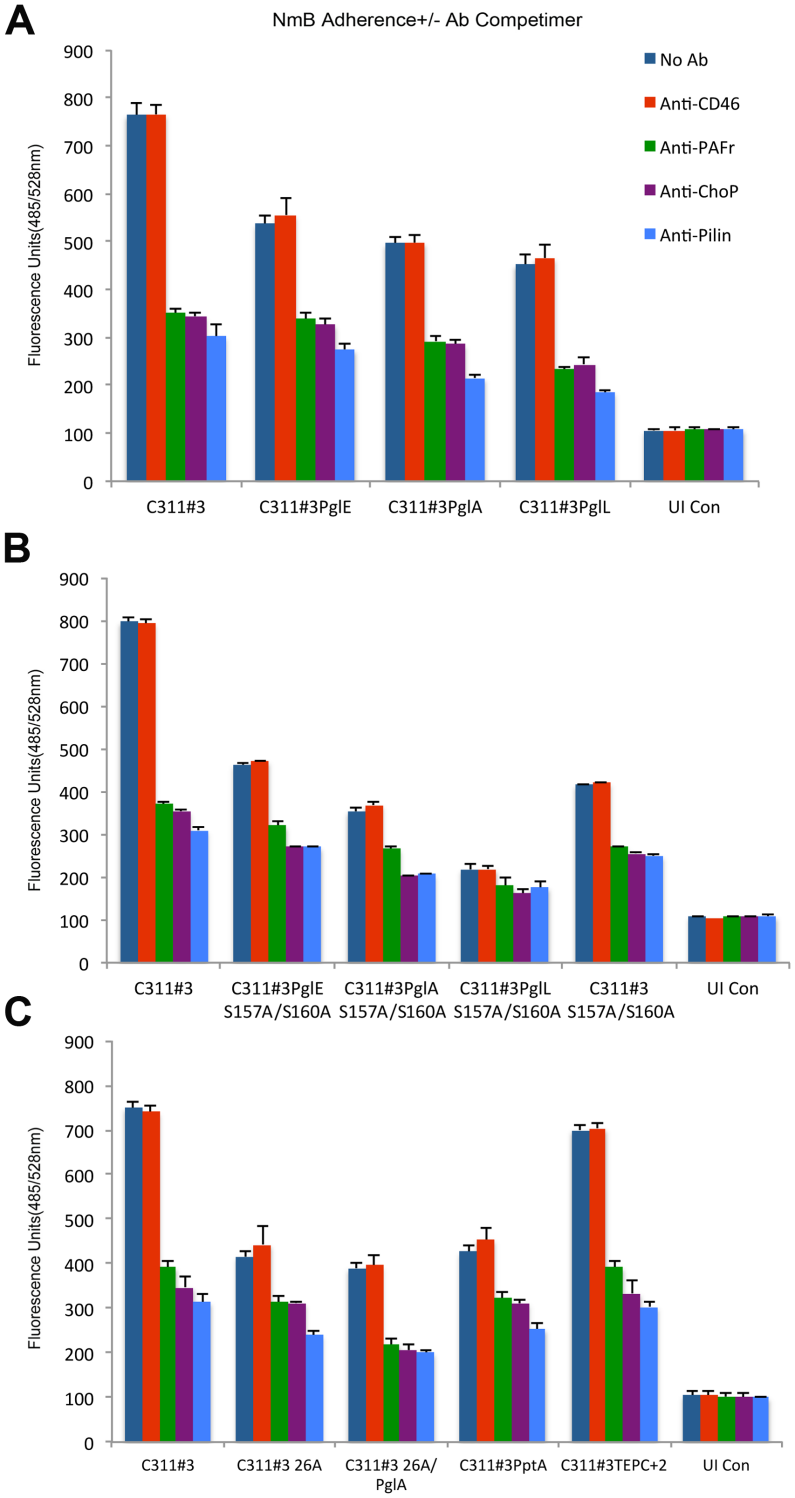
Cell association assays performed on a range of capsulate C311#3 mutants in the presence and absence of competimers. A fluorometric adherence assay, as described in the text, was used to determine the contribution of ChoP and glycan pilin modifications to a pilus-PAFr interaction on 16HBE14 cells. Antibody competimers were omitted (dark blue bars) or included in the assay and comprised anti-CD46 (red bars), -PAFr (green bars), -ChoP (purple bars), and -pilin (light blue bars). Arbitrary fluorescence units (y-axis) were recorded 30 minutes post-challenge of 16HBE14 cells with GFP-expressing C311#3 WT and mutant strains, as noted. UI Con - uninfected (control) 16HBE14 cells; (Panel A) C311#3 (WT, trisaccharide, 2 ChoP), PglE (*pglE* mutant, disaccharide, 2 ChoP), PglA (*pglA* mutant, monosaccharide, 2 ChoP), PglL (*pglL* mutant, no glycan, 2 ChoP); (Panel B) S157A/S160A (C311#3 site-specific mutant with a serine to alanine conversion generated at amino acids 157 and 160; trisaccharide, no ChoP); (Panel C) 26A (strain C311#326A, C311#3 natural variant, trisaccharide, no ChoP), 26APglA (C311#326A *pglA* mutant, monosaccharide, no ChoP), PptA (*pptA* mutant, trisaccharide, no ChoP), TEPC+2 (C311#3 natural variant with a serine to alanine conversion at amino acid 68; trisaccharide, 2 ChoP, hyper-reactive to TEPC-15 antibody). Adherence was significantly (P≤ 0.0001) impaired by the inclusion (vs. the omission of an antibody competimer) of anti-PAFr, -ChoP, or -pilin antibody competimers to each infection assay, whereas no significant (P≥0.2338) difference was observed when anti-CD46 antibody was included to block the association of meningococci with 16HBE14 cells. These data provide strong evidence that the initial contact of meningococci with human airway cells occurs via a pilus-PAFr interaction in which both the pilin glycan and ChoP play important contributory roles.

The intensity of PAFr- and pilus-corresponding bands in each Western Blot described above was highly variable among the C311#3 mutant and variant strains we examined. One explanation for this observation is that differences in the ability to adhere to 16HBE14 cells via the PAFr existed among the pilus glycosylation and ChoP mutants we examined. For example, in the anti-PAFr IP Western Blots; whereas an intense pilus-associated band was observed in the lane corresponding to the C311#3 WT infection, a band of moderate intensity was observed in the lane corresponding to infection with strains expressing pilus lacking either ChoP or glycan (Fig. 4C and S3). Therefore, to confirm and to quantitate data obtained above, as well as to determine the biological contribution of the glycan and ChoP pilus moieties to the PAFr-pilus association; we performed a fluorometric adherence assay. To this end, 16HBE14 cells were challenged with an expanded panel of C311#3 mutant and variant strains that varied in their expressed pilus structure (Tbl. S1). Antibodies capable of competing with *N. meningitidis* for PAFr binding were included or omitted from each assay (Fig. 5). These data strongly supported a role for both the pilus glycan and ChoP moieties in mediating *N. meningitidis* adherence to the PAFr in that: 1) anti-PAFr, -pilus, and –ChoP; but not anti-CD46; antibody competimers significantly decreased the association of C311#3WT with 16HBE14 cells, 2) glycan and ChoP C311#3 mutant and variant strains exhibited decreased adherence to 16HBE14 cells when compared to C311#3WT meningococci, and 3) the greatest decrease in adherence of meningococci to 16HBE14 cells occurred when the glycan and ChoP moieties both were absent from the pilus structure. Under these latter conditions, recorded fluorescence approached background levels, and adherence could not be further impaired by the presence of antibodies capable of competitively blocking a PAFr-pilus interaction.

**Figure 6:**
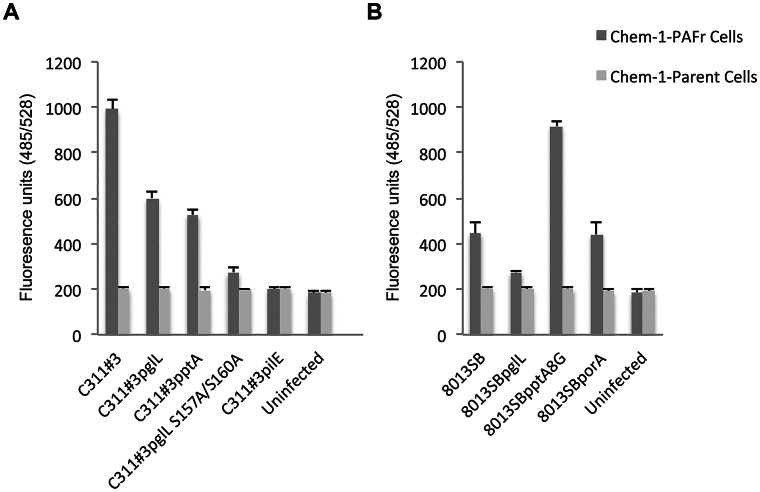
Comparative, quantitative, cell association assays performed using a PAFr-expressing cell line. A fluorometric adherence assay, as described in the text, was used to determine the contribution of ChoP and glycan pilin modifications to a pilus-PAFr interaction on Chem-1-PAFr cells or its parental cell line, Chem-1. Data shown were obtained simultaneously; however, for ease of discussion they are shown as separate panels. **A**) C311#3 and **B**) 8013SB WT, as well as their mutant derivatives, as noted, were used to challenge epithelial cells over-expressing the PAFr (*i. e.*, Chem-1-PAFr; dark grey bars) or cells devoid of the PAFr (*i. e.*, Chem-1; light grey bars). Adherence was recorded as arbitrary fluorescence units (FU) following a 30 min challenge and immunolabeling of meningococci with monoclonal (primary) antibody 2C3 and a FITC-conjugated secondary antibody, as described within the text. A significant difference in FU recorded was observed between C311#3 WT, C311#3*pglL*, C311#3*pptA*, and C311#3 (p≤0.001 for all comparisons) as well as when C311#3 and 8013SB strain Chem-1-PAFr cell infections were compared to infections performed using Chem-1 cells (p≤0.032 for all comparisons). Although there was a significant difference in the ability of strain 8013SB*pglL* (p≤0.037) and of strain 8013SB*pptA*8G (p≤0.003) to adhere to Chem-1-PAFr cells (when compared to 8013SB WT); the 8013SB*porA* strain was not significantly different from the WT strain (p≥0.321). There was no significant difference (p≥0.1) among any of the strains examined in their ability to adhere to Chem-1 cells, which do not express the PAFr, and only background levels of fluorescence were recorded for uninfected control cells.

To further examine the ability of meningococci to bind to the PAFr in the absence of extraneous factors, as well as to confirm the contribution of pili-linked glycan and ChoP PTMs in modulating PAFr binding; we performed comparative, quantitative fluorometric adherence assays using Chem-1 cells, which do not naturally express the PAFr, as well as Chem-1 cells in which the human PAFr was over expressed (Chem-1-PAFr). Chem-1 or Chem-1-PAFr cells were seeded to microtiter plates and meningococcal adherence was quantitated fluorometrically, as described in the Materials and Methods. Consistent with data obtained by IP, a significantly different (p≤0.001), glycan- and ChoP-dependent, hierarchical pattern of adherence was observed as: C311#3 WT≫C311#3*pglL*>C311#3*pptA*>>C311#3*pglL*S157/160A (Fig. 6). Only background levels of fluorescence (indicative of adherence) were detectable for uninfected cells or for Chem-1 cells that were infected in parallel with the Chem-1-PAFr cells (Fig. 6). Fluorescence units recorded for assays performed using C311#3*pilE* were only modestly significantly different (p≤0.04) than that recorded for uninfected Chem-1-PAFr cells, and there was no significant difference (p≤0.08) in C311#3*pilE* adherence to Chem-1-PAFr cells when compared to the Chem-1 parental cells (p≤0.08), demonstrating that the residual adherence observed occurred independently of a pilus-PAFr interaction. Thus, taken together, the above data strongly suggest that the PAFr serves as a receptor for pilus binding during meningococcal infections.

**Figure 7:**
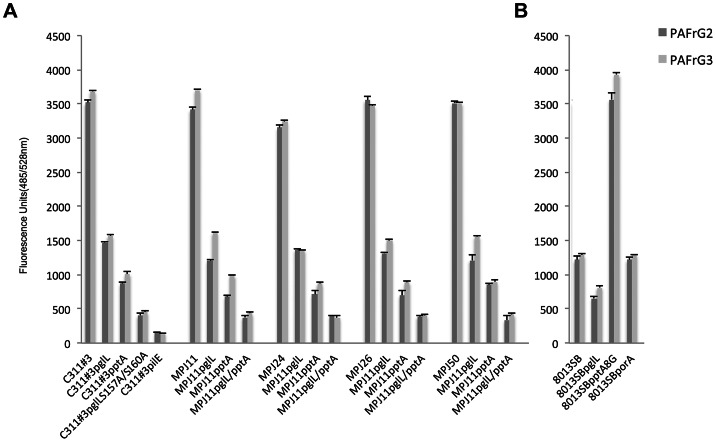
*N. meningitidis* pili-linked ChoP and glycan PTMs directly mediate an association with the PAFr. Purified pili and recombinant human PAFr were used to examine a direct pili-PAFR interaction by a modified ELISA, as described within the text. Pili used were isolated from a panel of *N. meningitidis* WT and mutant strains, as noted, and as are described in Tbl S1. Recombinant PAFr was coupled to the G-proteins, G_i_α_2_β_1_γ_2_ (PAFr-G2) or G_i_α_3_β_1_γ_2_ (PAFr-G3), which served as stabilizing agents. Data shown were obtained simultaneously; however, for ease of discussion they are shown as separate panels. **A**) WT and the mutant derivatives of strains C311#3, MPJ11, MPJ24, MPJ26, and MPJ50 served as a source for isolated pili. **B**) WT and the mutant derivatives of strain 8013SB WT, served as a source for isolated pili.

To directly assess the interaction of the pili-linked ChoP and glycan moieties in mediating an association with the PAFr, we next performed modified enzyme-linked immunosorbent assays (ELISAs) using purified components and as described in the “Materials and Methods”. In that the PAFr is not commercially available as an independent protein, two forms of stabilized PAFr were tested in our analyses: 1) recombinant human PAFr coupled to the G-protein, G_i_α_2_β_1_γ_2_ (PAFr-G2) as a stabilizing agent and 2) recombinant human PAFr coupled to the G-protein, G_i_α_3_β_1_γ_2_ (PAFr-G3) as a stabilizing agent. The wells of microtiter plates were lined with PAFr-G2 or -G3 to which pili isolated from a panel of *N. meningitidis* WT and mutant strains were added, as noted. Subsequent ELISA analyses again demonstrated a hierarchical pattern of pili adherence to PAFr-G2 and to PAFr-G3 that was consistent with that observed with the use of WT, mutant, and variant strains of C311#3 in IP (Fig. 4C and S3) and antibody inhibition (Fig. 5) assays using 16HBE14 cells, as well as in adherence assays performed using Chem-1-PAFr cells (Fig. 6A) The pilus-PAFr interaction was greatest for pili isolated from C311#3 WT (glycan+, ChoP+), and this interaction was diminished in those wells in which pili isolated from strains lacking the glycan and/or ChoP PTMs (*i. e.*, C311#3*pglL*, C311#3*pptA*, C311#3*pglL* S157A/S160A) were added (Fig. 7). To demonstrate that this interaction was not limited to strain C311#3, parallel assays were performed using the low passaged, clinically isolated; MPJ11, MPJ24, MPJ26, and MPJ50; WT and *pglL*, *pptA*, and *pglL*/*pptA* mutant strains. These assays yielded comparable data to that obtained with the C311#3 laboratory strains, indicating that the PAFr-meningococcal interaction is not a strain-specific phenomenon. Additionally, statistically significant differences were observed when comparing the use of the *pglL* mutant pili to the *pptA* mutant pili (p≤0.0001 for all meningococcal strains tested) as well as when comparing the use of *pptA* mutant pili to pili isolated from C311#3 S157A/S160A (p≤0.0001) or from the “MPJ” *pglL/pptA* double mutant strains (p≤0.0001 for all strains tested), demonstrating that synergy exists between the glycan and ChoP moieties in mediating adherence to the PAFr. Further, in that comparable data were obtained with the use of PAFr-G2 or PAFr-G3 (Fig. 7), in which distinct G-proteins were used to stabilize recombinant human PAFr, the interaction observed between pili and PAFr-G2 or -G3 can be concluded to result from a specific interaction with the PAFr and not the stabilizing G-protein component.

To determine whether strains that express the *pptAG11* form of *pptA* (and, therefore, express the inactive PtaAGly^+1^ protein) are capable of an interaction with the PAFr in the rare event that they under-go phase variation to produce an active form of PptA and, thus, add ChoP to their pili, we genetically modified strain 8013SB WT to contain *pptA8G* (strain 8013SB*ppt*A8G) and, thus, express an active form of PptA (Fig. 1). Strains 8013SB WT, 8013SB*pglL*, and 8013SB*pptA*8G were then examined for their ability to interact with Chem-1 and Chem-1-PAFr cells, as described above. Although each of the 8013SB WT and mutant strains examined displayed a PAFr-dependent association with the Chem-1 cells, the presence of ChoP on strain 8013SB*pptA8G* pili resulted in a greater than 2-fold increase in the ability of this strain to adhere to Chem-1-PAFr cells, when compared to 8013SB WT bacteria. Additionally, although 8013SB WT (ChoP−) bacteria were significantly less adherent to Chem-1-PAFr cells, than that observed for C311#3 WT (ChoP+) bacteria (p≤0.0001), adherence levels for strain 8013SB*pptA8G* (ChoP+) were not significantly different (p≥0.081) from C311#3 WT (Fig. 6B). Support for these data is obtained by the inclusion of pili isolated from 8013SB, and its derivatives, in the ELISA assays described above, in which the pili-PAFr-G2 or -PAFr-G3 interaction was examined (Fig. 7B). A direct interaction between pili isolated from these strains and the PAFr is readily observed, as is the involvement of both ChoP and the pilin-linked glycan in this interaction. In this strain, the *pptA* gene is heterologously expressed from the *porA* promoter, resulting in the deletion of the *porA* gene, and the loss of PorA expression. A control strain, in which the *por*A gene was independently inactivated, revealed that the absence of PorA had no impact on 8013SB adherence to Chem-1 or Chem-1-PAFr cells. Similarly, the absence of PorA did not effect the ability of 8013SB-derived pili to adhere to PAFr-G2 or -G3 by ELISA. In all experiments, the 8013SB*porA* mutant strain behaved as did the 8013SB WT strain, indicating that data obtained with the use of strain 8013SB*pptA8G* were not the result of a genetic aberration introduced in generating the latter strain.

Thus, collectively, the above data provide strong support for a key role for the PAFr in mediating adherence of meningococci to bronchial epithelial cells by a mechanism in which the glycan and ChoP moieties present on the core pilus structure act synergistically.

## Discussion

The presence of ChoP on pilin is subject to phase variation, and this results from alterations in the length of the homopolymeric guanine tract in *pptA*, the gene encoding the transferase responsible for linking ChoP to pilin [17]. The presence of surface exposed, phase variable ChoP is not unique to *Neisseria*. For example, ChoP is a covalently linked, post-translational modification found on the surface of predominate respiratory pathogens, *e. g. H. influenzae* [23], *S. pneumoniae* [19], and *Pseudomonas aeruginosa* [35] [24] [36] for which a role in mediating adhesion and invasion to host epithelia is described for ChoP. Additionally, C-reactive protein (CRP) present in blood has the potential to bind to ChoP that, in turn, can result in complement-mediated killing of *H. influenzae* bearing ChoP on their surface [22]. In this scenario, expression of ChoP would serve as a selective disadvantage to the microorganism. This has led to the idea that phase variation of ChoP provides one mechanism by which invasive pathogens adapt to a transitional lifestyle that occurs with respect to colonization of the respiratory mucosa and, subsequent, survival during systemic infections [22]. We could not demonstrate CRP/complement-mediated killing of strain C311#3 using the same methodology as described for *H. influenzae* (20); however, Casey *et al.* (2008) have shown ChoP-dependent opsonophagocytic killing of strain C311#3, suggesting that expression of ChoP by meningococci may be disadvantageous in some environments. As ChoP is clearly important in the pathobiology of other respiratory pathogens, we sought to analyse its role in pili-related functions in *N. meningitidis*.

When this work commenced, although neisserial pilin was known to be modified with ChoP, the location of ChoP on *N. meningitidis* pilin was undefined. Therefore, we initiated our investigation by asking whether ChoP was surface exposed in *N. meningitidis* strain C311#3. Colony immunoblotting performed using the ChoP-specific antibody, TEPC-15, revealed that ChoP was indeed surface exposed in this strain. However, they also revealed that a small number of colonies were hyper-reactive to antibody TEPC-15, hinting that ChoP may be aberrantly presented or exposed in these hyper-reactive bacterial colonies. Additional Colony Blot analysis of isolated TEPC-15 hyper-reactive C311#3 variants confirmed that the accessibility of ChoP increased as the length of the pilin-linked glycan was shortened. However, this hierarchy of ChoP exposure was only seen for pili in its native, polymerised, state and not for denatured pilin examined by western blotting. The presentation and exposure of ChoP on meningococcal pili has the potential to affect the receptor-ligand interactions with host cells. Therefore, we sought to investigate the underlying mechanism(s) for TEPC-15 hyper-reactivity by sequencing the *pilE* and phase variable pilin glycosylation genes of C311#3 strains TEPC+1, +2, and +3. Two of these TEPC-15 hyper-reactive variants, C311#3 TEPC+1 and C311#3 TEPC+2, displayed changes in their respective *pil*E sequences. These sequence variations occurred near the site of PilE glycosylation or near the 3′ end of the *pil*E gene. The other variant, C311#3 TEPC+3, had a frame shift in the phase variable pilin glycosylation gene, *pgl*E. Collectively, these data provided evidence that, in addition to pilin amino acid changes, alterations to the trisaccharide structure also had the potential to influence the accessibility of ChoP on pili.

In determining the site of ChoP modification to *N. meningitidis* pili, we hypothesised that ChoP would be O-linked to a serine residue because 1) ChoP is shown to be O-linked to sugar residues on the surface of other bacteria [17], 2) serine and threonine residues are preferred sites for O-linked (protein) modification, 3) the primary sequence of meningococcal pilin contains a DASDAS motif at its C-terminus that could serve as a potential site(s) for O-linked ChoP addition, and 4) sequence variations occurring near the site of pilin glycosylation or near the 3′ end of the *pil*E gene altered the accessibility of antibody TEPC-15 to pilin-linked ChoP. To test this hypothesis, we initially targeted seven serine residues (Ser34, Ser45, Ser68, Se69, Ser70, Ser157 and Ser160) for conversion to alanine residues by site-directed mutagenesis. These seven serine residues were chosen for mutagenesis as they were located near the O-linked glycosylation site (Ser63) in the mature pilus fibre of *N. meningitidis*, and during the course of our investigations, it was reported that ChoP is linked to Ser 68 in *N. gonorrhoeae* strain MS11 (Hegge et al., 2004). In contrast to what was observed for *N. gonorrhoeae*, none of our *N. meningitidis* mutants, including a mutant harbouring a S68A mutation, showed the loss of ChoP.

LC-MS/MS and mutagenesis analyses defined the location of ChoP as the pilus C-terminus. Upon consideration of the relative position of the ChoP and glycan modifications of pilin, it was difficult to resolve how the O-linked pilin glycan present on Ser63 could affect ChoP accessibility (on Ser157 and Ser160); however, these data are consistent with our present model of the meningococcal pilus quanternary structure. That is, we have presented a model in which the pilin-linked glycan and ChoP moiety are remotely associated in the pilin monomer but reside in proximity in the context of the polymeric pilus fiber, and the surface accessibility to ChoP mediated by glycan structure described above may have functional consequences in host-pathogen interactions.

In *N. gonorrhoeae* MS11, Ser68 is reported to be the site for ChoP addition [37], which is in proximity to the Ser63 site of glycan linkage. The same group has also reported a phosphoethanolamine modification at Ser156 of MS11 pilin [38]. In contrast, *N. meningitidis* C311#3 pilin has ChoP on both Ser 157 and 160. Changing both of these residues to alanine resulted in the complete loss of TEPC-15 immunoreactivity. This suggests that the C-terminus of meningococcal pilin is the only site of ChoP modification, and further, modification by phosphoethanolamine was not observed in our study. This fundamental difference in meningococcal and gonococcal PTM by phosphate moieties may reflect the different lifestyles associated with these two *Neisseria spp.* Support for this idea is derived from studies examining ChoP expression by *H. influenzae* in which it is demonstrated that changing the site of ChoP addition on the surface of this bacterium corresponds to differential CRP-mediated bactericidal activity [39]. In this regard, *H. influenzae* seems to have developed methods to balance the beneficial and detrimental effects of ChoP exposure by varying both the presence/absence of ChoP displayed on its surface as well as the specific site of ChoP addition. In contrast to the systemic disease caused by *N. meningitidis*, in which CRP would be expected to play a role, *N. gonorrhoeae* is rarely associated with systemic infection.

Colonization of the nasopharnyx precedes meningococcal disease and requires adhesion to the mucosal epithelium. Previous invasion data had implicated CD46 as the pilus receptor for the pathogenic *Neisseria* [40]. However, CD46 is basolaterally located on the mucosal epithelium [40] and an increasing body of evidence has ruled out a role for this host cell molecule as serving as the initial pilus receptor mediating adherence to host cells by *N. gonorrhoeae* [41] [42] [43] [44]. Although several pilus receptors are described for the gonococcus [45] [42] [43], the pilus-host cell first point of contact in the airway has not been identified for *N. meningitidis*. Given that the ChoP moiety on platelet activating factor serves as the primary ligand for the PAFr and that several respiratory pathogens are able to bind to the PAFr through an interaction that involves surface exposed ChoP. We hypothesized that a pilin-linked ChoP-PAFr interaction could exist as one mechanism mediating *N. meningitidis* colonization of the human airway. Evidence we provide to support this hypothesis include data obtained from confocal microscopy and IP assays in which we observed co-localization of meningococci or meningococcal pilin with the PAFr on 16HBE14 SV40-transformed, human bronchial epithelial cells as well as on infected human bronchial tissue. We further show that the presence of anti-CD46 antibody competimers had no effect on the adherence of C311#3 WT and mutant bacteria to 16HBE14 cells; whereas, parallel assays performed using anti-PAFr, -pili, and -ChoP antibody competimers resulted in a significant (P≤0.001) decease in meningococcal adherence. Additionally, we demonstrate *N. meningitidis* adherence to human PAFr-expressing Chem-1 cells as well as the direct association of pili isolated from a panel of laboratory and low passage, clinically isolated meningococci to stabilized recombinant human PAFr (PAFr-G2 and PAFr-G3). Taken together these data provide strong evidence that the PAFr serves as a key receptor mediating the initial, pilus-dependent, interaction of *N. meningitidis* with the airway epithelium. Furthermore, evidence to indicate that *both* the pilin-linked glycan and ChoP contribute directly to PAFr-mediated adherence to 16HBE14 cells are found in the following observations: (1) ChoP dependent adherence is directly related to the length of the pilin-linked glycan due to altered accessibility (trisaccharide; ChoP+ (C311#3 WT)≫disaccharide; ChoP+ (C311#3*pglE*)>monosaccharide; ChoP+ (C311#3*pglA*)>no glycan; ChoP+ (C311#3*pglL*)) and (2) glycan dependent adherence is further decreased (but not abolished) when ChoP is absent ((trisaccharide; ChoP+) C311#3>>>(trisaccharide; ChoP−) C311#3 S157A/S160A). This same hierarchy of interaction was observed in 4 independent *N. meningitidis* clinical isolates. To our knowledge, this situation, in which dual post-translational modifications of a bacterial adhesion mediate interactions with the host cell receptor, is unique in biology.

The pivotal steps in the transition of some *N. meningitidis*-infected individuals from a state of harmless, asymptomatic carriage to rapidly progressing, often fatal, sepsis are poorly understood. A series of recent studies focusing on the interactions of meningococci with endothelial cells have reported that type IV pili can mediate adhesion to brain endothelial cells allowing bacteria to cross the blood-brain barrier via ß2-adrenoceptor/ß-arrestin signalling pathway [46] [47]. This signalling pathway does not occur in adhesion to epithelial cells [48]. In particular, Chamot-Rooke *et al.* (2011) reported that the addition of phosphoglycerol to meningococcal pilin by PptB plays a role in initiating disseminated infections. These authors propose that, upon contact with the human airway, *pptB* is up-regulated; pilin is decorated with phosphoglycerol, that then causes the dissociation of bacterial colonies with the airway epithelium and, in turn, disseminated infection. The phosphoglycerol PTM study described above raises several key mechanistic questions (for commentary see [49]), and focuses on a PTM distinct from those described herein. However, both studies address pili PTM-mediated host-pathogen interactions, so it is import to consider the key differences between our investigations and those of Chamot-Rooke *et al.* (2011). Our investigations were aimed at identifying the crucial, *initial*, events mediating meningococcal adherence at the airway interface, including the identification of the previously unknown receptor involved in this process. Our on-going studies are focused on elucidating the events mediating pathogenesis following that particular mechanism of adhesion. The Chamot-Rooke *et al.* (2011) study involved the use of a human endometrial cell line for adhesion assays. This cell type is not relevant to the initial contact step occurring within the airway. For initial attachment studies, we used physiologically relevant human bronchial cells as well as human bronchial tissue. Secondly, we identified the key, initial, receptor in cell attachment, whereas Chamot-Rooke *et al* (2011) did not characterise the human cell receptor that mediates adherence observed in their studies. Rather, they focused on the bacteria to bacteria interaction mediated by pili (pili bundling) with the distinct, *pptB* mutant generated, phosphoglycerol PTMs phenotype. Further, it is important to note that in strain 8013SB (used in the Chamot-Rooke *et al*, 2011, study) the PptA ChoP transferase that is expressed from *pptA11G* is the inactive PptAGly^+1^ form (see Fig. 1). Strain 8013SB is atypical of most meningococcal clinical isolates in that it does not express ChoP on pili. Therefore, the rapid and early engagement of the PAFr described herein would not have been be possible to observe in the strain (8013SB) used in the Chamot-Rooke *et al* study, or, indeed, in the other endothelial cell signalling studies noted above, which also used strain 8013SB [46], [47], [48]. However, by altering the homopolymeric “G” region of this same strain such that a ChoP+ phenotype would be expressed (*i. e.*, strain 8013SB*ppt*A8G), we have shown that adherence to Chem-1-PAFr cells as well as PAFr protein is increased greater than 2-fold. Nevertheless, it is presently not clear if a meningococcal ChoP-PAFr interaction would result in the complex bacterial detachment phenotype observed by Chamot-Rooke *et al* (2011). Similarly, it is not clear if the pili bundling phenotype (suggested to result from the negative charge associated with the phosphoglycerol PTM in strain 8013) would, indeed, occur with strain 8013SB were it capable of naturally undergoing pilin ChoP (positive charge) PTM, as is true for the vast majority (>94%) of meningococcal clinical isolates.

Although pili are critical to *N. meningitidis* colonization in being able to promote the adherence of capsular, as well as acapsular organisms; the host surface molecule recognized, and bound, by pili had previously remained elusive despite extensive study. In this regard, we have provided the first evidence demonstrating a role for both the pilin-linked glycan and ChoP as important contributors in the adherence of *N. meningitidis* to human bronchial epithelial cells. However, of greater importance is our identification of the PAFr, a G-protein coupled receptor, in mediating the earliest contact with human bronchial cells and tissue in that the immunomodulatory properties of the PAFr are well-characterized and include a specific role in promoting polymicrobial-induced sepsis [50] [51]. Whether the engagement of the PAFr within the human airway by the pilin glycan and/or ChoP results in signalling events consistent with an acute inflammatory response remains to be determined. Similarly, it is not known if the expression of ChoP and/or a particular pilin glycan are predominate in *N. meningitidis* isolated from the human airway, human blood, or human cerebral spinal fluid. These and other unanswered questions are the basis for on-going studies in our lab.

## Materials and Methods

### Bacterial strains and media

Meningococcal strains used in this study are listed in Tbl S1. Meningococcal strains were grown on Brain Heart Infusion (BHI)-1% agar-10% (both from Oxoid) - Levinthals Base medium at 37°C with 5% CO_2_ for 16–18 hrs. *Escherichia coli* strain DH5α was used to propagate cloned plasmids and were grown at 37°C in Lauria-Bertani (LB) broth supplemented with either ampicillin (100 µg/ml) or tetracycline (5 µg/ml).

### Genetic manipulations and analyses

All primers used for the following procedures are listed within Tbl. S2. The plasmid, pGEM*TetMBpilE::His/lpxC* [52], was modified using splice overlap PCR [53] to construct the different serine mutants. The upstream region of *pilE* was amplified using the primer PilE-NotI and the relevant reverse primer. The downstream region was amplified using the Tet-HindIII primer and the relevant forward primer (incorporating the desired change). The mutated full-length product was then amplified by a reaction consisting of an equal mix of upstream and downstream DNA and by using the primers PilE-NotI and Tet-HindIII. This amplicon was digested using the restriction endonucleases *Not*I and *Hind*III and ligated into the plasmid backbone of *Not*I- and *Hind*III-digested pGEM*TetMB::lpxC* [52]. A FLAG-tagged *pilE* was constructed by fusing the FLAG sequence to *pil*E using the primers PilE-NotI and FLAG-XhoI. The primer, FLAG-XhoI, comprises the FLAG-tag and an *Xho*I digestion site extension that allows in-frame tag incorporation at the C-terminus of *pil*E, and, therefore, does not interfere with secretion of the mature pilin peptide. Following digestion, the resulting *pil*E::FLAG DNA fragment was directionally ligated into pGEM*TetMBlpxC*. The resulting plasmid was sequenced to ensure the desired change had occurred and transformed into acapsular *N. meningitidis* C311#3, as previously described [14].

The *pptA* gene in *N. meningitidis* C311#3 was inactivated by the insertion a tetracycline (*tetM*) cassette as described in [17]. The *pptA* genes from C311#3 and 8013SB were amplified by primers (pptA_*EagI* and pptA_*NcoI* Table S2 and cloned into the *EagI* and *NcoI* sites in plasmid pCO14k [54] which can be used for the heterologous expression of genes from the *porA* locus [55]. The plasmids of *pptA* gene with various polyG numbers were constructed by inverse PCR using the primers described in Table S2. The various pCO14k*pptA* plasmids were linearized and transformed into the C311#3*pptA::tet* and 8013SB.

### Pilin purification

C311#3*pilE*::*FLAG* were grown overnight on BHI agar, harvested into TE buffer and heat-killed for 1 h at 56°C. The bacterial cells were lysed by a French-press (5 times, 1000 psi) after which the lysate was centrifuged (14,000 *g*, 30 min, 4°C). The supernatant was then loaded onto an affinity gel column with ANTI-FLAG M2 Affinity Gel (Sigma). FLAG-tagged pilin was purified per the manufacturer's instructions (Sigma). Column purified, FLAG-tagged pilin was then processed for spectrometric analysis, as outlined within the text. In separate experiments, pili from WT and mutant meningococci were isolated as described by Power *et al.* [56] and analysed by western blotting, also as outlined within the text.

### In-solution pilin digestion and LC-MS/MS

Purified pilin was reduced and then alkylated with 50 mM dithiothreitol and 100 mM iodoacetamide, respectively. Four volumes of 1∶1 acetone∶methanol were added, incubated (−20°C for 16 h) and centrifuged (18,000 rcf, 10 min) to collect alkylated pilin. This protein was then dried at room temperature (RT) before resuspension in 50 µL of 50 mM NH_4_HCO_3_ and digestion (37°C, 16 h) with 1 µg trypsin (Sigma). Digested pilin peptides then were analysed by LC–ESI/MS/MS using an API QSTAR Pulsar i LC/MS/MS system (Applied Biosystems). Samples were separated on a ZORBAX SB-C18 5 µm, 150×0.5 mm column previously equilibrated with 5% acetonitrile-0.1% formic acid in water, after which they were eluted over 45 min using a gradient from buffer A (0.1% formic acid) to buffer B (90% acetonitrile with 0.1% formic acid). Analyst QS 1.1 software was used to manually examine LC–MS and MS/MS data for the presence of predicted peptides and for ChoP-modified peptide.

### Confocal microscopy

WT, mutant, and derivative C311#3 strains were transformed with the green fluorescent protein (GFP) expressing plamid pCmGFP [57], as previously described [58]. Confocal microscopy of GFP-expressing C311#3-infected and uninfected 16HBE14, SV40-transformed, human airway epithelial cells [59] was conducted as previously described [23] following immunolabeling with rabbit anti-PAFr immune sera, Alexa 647:Goat anti-rabbit secondary antibody, and staining with CellTracker Red (Invitrogen). The 16HBE14 cell line was originally derived by SV-40 transformation of a polyclonal population of cells obtained from human bronchial epithelium and thus is heterogeneous.

A Zeiss 510 Laser scanning confocal microscope located at the Central Microscopy Research Facility (University of Iowa, Iowa City USA) was used to evaluate co-localization of C311#3 with the PAFr.

Immunolabeling of frozen infected bronchial tissue sections was performed as described by Edwards *et al.* (2000). Primary antibodies used for immunolabeling were specific for the PAFr (H-300, rabbit) or *N. meningitidis* PIB (pS-20, goat; both from Santa Cruz Biotechnology, Santa Cruz, CA). Rhodamine- or FITC-conjugated secondary antibodies (Jackson ImmunoResearch Laboratories, West Grove, PA) were applied to tissue sections, cell monolayers, and bacteria, as noted. Omission of the primary antibodies served as a negative control for non-specific labelling and autofluorescence. Immunolabeled tissue cryosections and cell monolayers were viewed using the Zeiss 510 Laser Scanning Confocal viewing system located at the Research Institute at Nationwide Children's Hospital (Columbus, OH).

### Immunolabeling of pilin and pilin-associated complexes

Western or colony blotting was performed using standard protocols and the following primary antibodies: 1) mouse anti-ChoP, TEPC-15, (Sigma, St. Louis MO), 2) mouse anti-pilin, SM1, [33], 3) rabbit polyclonal anti-pilin, or 4) goat anti-PAFr antibody, C-20 (Santa Cruz Biotechnologies, Santa Cruz, CA). Colorimetric/chemiluminescent detection of pili or pilin-associated immune complexes was obtained, as noted, following the application of a phosphatase- or a peroxidase-conjugated secondary antibody and the addition of the appropriate enzyme substrate. In separate experiments; bacterial colonies, purified pili, as well as anti-PAFr or anti-pilin immunoprecipitates served as substrates for immunolabeling. For colony immunoblotting, overnight cultures of meningococci were subcultured on BHI agar and plated to allow single colony formation. Immunoprecipitation was performed as described by Wen *et al.* [60]. Rabbit immune serum to C311#3 pilus or goat anti-PAFr antibody, C-20, were used to capture immune complexes. C311#3-infected, 16HBE14 cell lysates in which the anti-PAFr or –pilus primary, or agarose-conjugated secondary, antibodies were omitted (negative controls) were treated in parallel with uninfected and infected cell lysates to which antibodies had been added.

### Fluorometric adherence assays

The ability of WT and mutant *N. meningitidis* strains to adhere to the PAFr was determined using modified fluormetric ELISAs. 16HBE14, Chem-1, or PAFr-expressing Chem-1 (Chem-1-PAFr; EMD Millipore, Billerica, MA) cells were used to assess the ability of bacteria to adhere to the PAFr on a cellular surface. 16HBE14 cells were passed to 96-well microtiter plates and allowed to grow to confluence in Airway Medium [61]. Chem-1 and Chem-1-PAFr cells were seeded to microtiter plates at 10^4^ cells per well using (DMEM-based) medium supplied by the manufacturer. At least 24 h before use in infection studies, culture media were replaced with antibiotic- and serum-free medium. For antibody competiton assays, anti-PAFr [23], -ChoP (XC10), or - pilin rabbit immune serum was added to select microplate wells to yield a final antibody dilution of 1∶50. Anti-CD46 antibody, H-294, (20 µg/ml, Santa Cruz) served as an arbitrary antibody competimer control. Immediately following antibody addition, GFP-expressing meningococci were used to challenge 16HBE14 cells at a multiplicity of infection of 100. Blank wells, devoid of human cells, also were inoculated with bacteria and served as controls for potential fluorescence intensity differences among the bacterial strains examined. Chem-1 and Chem-1-PAFr cells were challenged with 10^6^ *N. meningitidis* WT or mutant (non-GFP-expressing) bacteria per well, as noted. For either assays, infections were stopped after 30 min by the removal of the infection medium, extensively rinsing the cell monolayers with phosphate buffered saline, and cell fixation. Following fixation, were noted *N. meningitidis* were immunolabeled using monoclonal antibody 2C3, which recognizes the conserved H.8 outer membrane protein of the pathogenic *Neisseria*, and a FITC-conjugated secondary antibody. Infected, control 16HBE14 cell assays (devoid of antibody competitors) and uninfected, control cell assays (with antibody competimers) were treated in parallel with competitive antibody inhibition assays. Uninfected and infected, Chem-1 and Chem-1-PAFr cells were, similarly, treated in parallel within the confines of the same microtiter plate. Fluorescence intensity, corresponding to bacteria adherence, was recorded using a Synergy HT Multi-mode Microplate Reader (BioTek Instruments, Winooski, VT). Each assay set was performed in triplicate on 3 separate occasions. A Kruskal-Wallis k-sample analysis of variance was used to determine the statistical significance of the calculated mean adherence (recorded as fluorescence units) for each of the bacterial strains examined under each assay condition. No significant difference (p≤0.02) was observed among the bacteria inocula used to challenge 16HBE14 cells, as determined by a Student-t Test.

### Determination of a direct *N. meningitidis* pili-PAFr interaction

Modified ELISAs were also used to examine the direct interaction of *N. meningitidis* pili with the PAFr. Purified PAFr, as a single protein entity, is not commercially available; therefore, 2 sources of stabilized human PAFr were comparatively evaluated for their ability to interact with meningococci or isolated meningococcal pili. These comprised recombinant PAFr coupled to the G-proteins G_i_α_2_β_1_γ_2_ (PAFr-G2) or G_i_α_3_β_1_γ_2_ (PAFr-G3; Axxora, Farmingdale, NY).

In separate studies, 96-well microtiter plates were coated with 1 µg PAFr-G2 or PAFr-G3. Plates were then incubated with 10 µg pili isolated from each meningococcal strain (as described in 59). Plates were washed (6×) before immunolabeling an anti-pilin (rabbit) primary antibody and a FITC-conjugated secondary antibody. The PAFr-*N. meningitidis* pili interaction was subsequently recorded as arbitrary fluorescence units (FUs) at 485 nm excitation/528 nm emission using a Synergy HT multimode plate reader. Assays were performed in quadruplicate on three separate occasions. Statistical significance of the data obtained was determined using paired Student-t Tests.

## Supporting Information

Figure S1 **PilE gene sequence alignment of high TEPC-15 reactive colonies.** The amino acid sequence, and sequence conservation surrounding the post-translational modifications of pilin, in *Neisseria* are shown. Superscript numbers represent the amino acid number in the mature pilin. The arrow indicates the site of trisaccharide addition. The variable structures are shown in the red boxes.
(TIF)

Figure S2 **Analysis of FLAG-tagged purified pilin.** To undertake structural studies on ChoP modification of pilin, it is necessary to express and purify large quantities of pure, soluble pilin from *N. meningitidis*. In this study, pilin of *N. meningitidis* was purified using a Flag-tag-based system. Western Blot analysis of purified pilin was performed to confirm post-translational modifications occurred to purified pilin in the context of a FLAG-tag. To this end, we used anti-pilin polyclonal sera (that binds C311#3 pilin), monoclonal antibody TEPC-15 (that binds the phosphorylcholine structure), anti-trisaccharide polyclonal sera (that binds the trisaccharide glycan structure), and an anti-FLAG M2 monoclonal antibody (that binds the FLAG-tag sequence at the C-terminus of purified pilin). Results from this analysis indicated that FLAG-tag expression and the purification process did not interfere or disrupt the post-translational modification of the recombinant tagged pilin protein with ChoP or the glycan.
(TIF)

Figure S3 **Immunoprecipitation of PAFr and pili from various C311#3 mutants and variants.** Immunoprecipitation followed by Western Blot analysis demonstrates that meningococci bind to the PAFr via an interaction involving both the pili-linked ChoP and glycan modifications. Following a 15 minute challenge of 16HBE14 human bronchial epithelial cells, captured anti–pilin or –PAFr immunoprecipitates were transferred to a solid support medium and then subjected to western blotting with (A) anti-PAFr and (B) anti-pilin antibodies, as outlined in the text. The image shown was obtained from a single blot resulting from each immunoprecipitation condition; however, the centre portion of each blot (not relevant to this study) was deleted. No 1°Ab - the primary antibody was omitted from the initial immunoprecipitation capture step; No 2° Ab - the secondary, agarose-conjugated antibody was omitted from the immunoprecipitation assay; UI – uninfected cells. A panel of *N. meningitidis* C311#3 strains were evaluated for their ability to adhere to 16HBE14 cells via a pills-mediated mechanism and are indicated across the top panel. Pilin PTM for each strain are as follows: C311#3 WT (trisaccharide; ChoP+), C311#3*pilE* (pilin−; glycan−; ChoP−), C311#3*pglE* (disaccharide; ChoP+), C311#3*pglA* (monosaccharide; ChoP+), C311#3*pglL*(glycan−; ChoP+), C311#3 26A*pglA* (monosaccharide; ChoP−), C311#3 26A (trisaccharide; ChoP−), C311#3*pptA* (trisaccharide; ChoP−), C311#3*pglE*S157A/S160A (disaccharide; ChoP−), C311#3*pglA*S157A/S160A(monosaccharide; ChoP−), C311#3*pglL*S157A/160A (glycan−; ChoP−).
(TIF)

Movie S1 **The relative positions of phosphorylcholine and glycosylation post-translational modifications modelled on pilin subunit in** ***Neisseria meningitidis*** **.**
(MOV)

Movie S2 **The relative positions of phosphorylcholine and glycosylation post-translational modifications of adjacent pilin subunits on pilus fibre in** ***Neisseria meningitidis*** **.**
(MOV)

Table S1 **Bacteria strains used.**
(DOCX)

Table S2 **List of primers.**
(DOCX)

Table S3 **PptA polymeric G screening of** ***Neisseria*** **MLST typed and clinical isolate strains.**
(DOCX)
