# Supplementary figures and images for: Dual Pili Post-translational Modifications Synergize to Mediate Meningococcal Adherence to Platelet Activating Factor Receptor on Human Airway Cells
Source: PLoS Pathog. 2013 May 16;9(5):e1003377. doi: 10.1371/journal.ppat.1003377 (PMC3656113; doi:10.1371/journal.ppat.1003377)

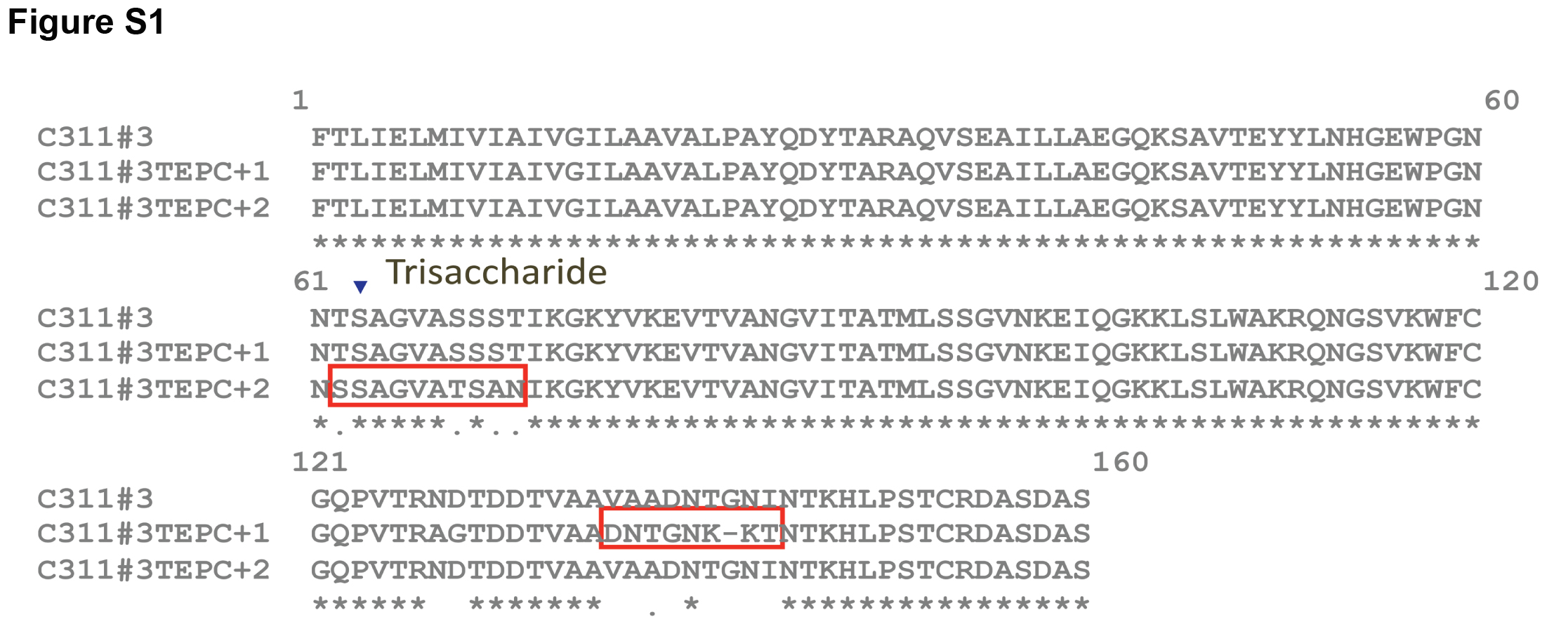

Supplement: Figure S1 — PilE gene sequence alignment of high TEPC-15 reactive colonies. The amino acid sequence, and sequence conservation surrounding the post-translational modifications of pilin, in Neisseria are shown. Superscript numbers represent the amino acid number in the mature pilin. The arrow indicates the site of trisaccharide addition. The variable structures are shown in the red boxes. (TIF) [file ppat.1003377.s001.tif]

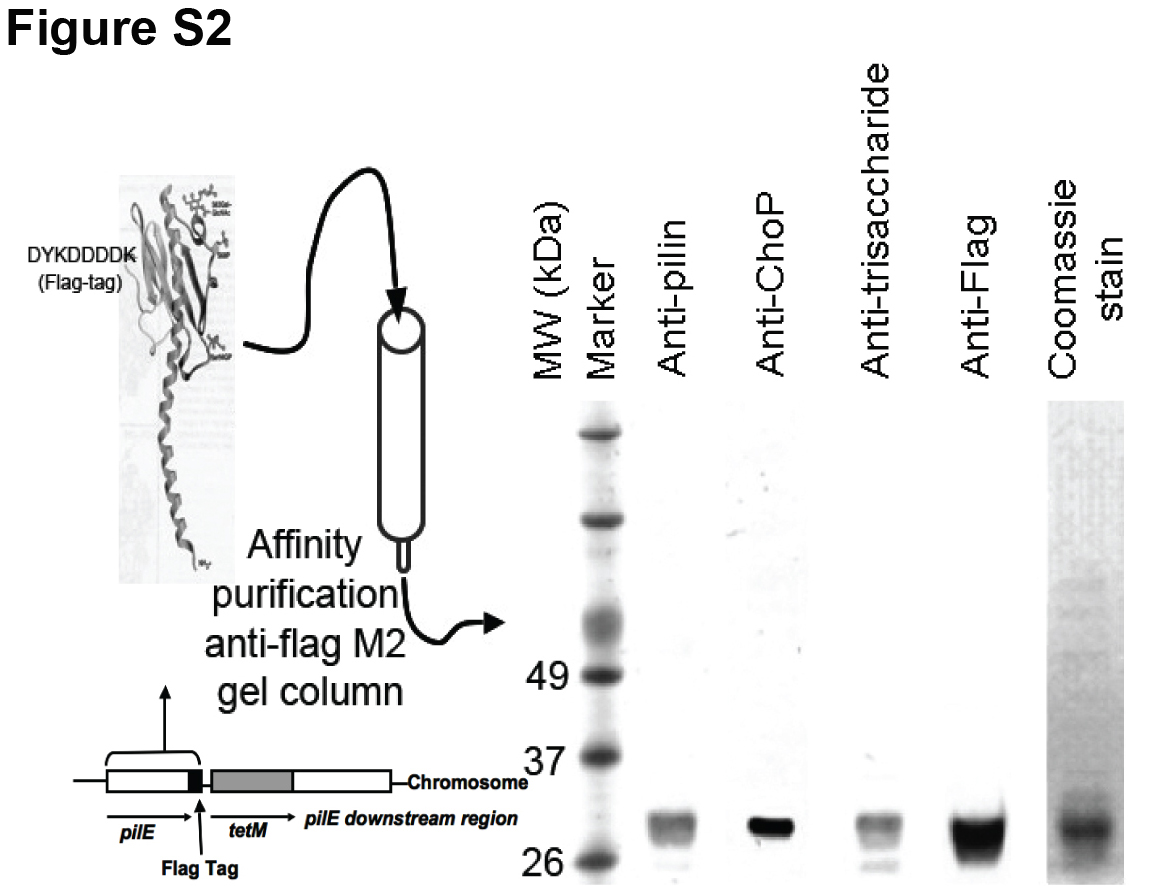

Supplement: Figure S2 — Analysis of FLAG-tagged purified pilin. To undertake structural studies on ChoP modification of pilin, it is necessary to express and purify large quantities of pure, soluble pilin from N. meningitidis. In this study, pilin of N. meningitidis was purified using a Flag-tag-based system. Western Blot analysis of purified pilin was performed to confirm post-translational modifications occurred to purified pilin in the context of a FLAG-tag. To this end, we used anti-pilin polyclonal sera (that binds C311#3 pilin), monoclonal antibody TEPC-15 (that binds the phosphorylcholine structure), anti-trisaccharide polyclonal sera (that binds the trisaccharide glycan structure), and an anti-FLAG M2 monoclonal antibody (that binds the FLAG-tag sequence at the C-terminus of purified pilin). Results from this analysis indicated that FLAG-tag expression and the purification process did not interfere or disrupt the post-translational modification of the recombinant tagged pilin protein with ChoP or the glycan. (TIF) [file ppat.1003377.s002.tif]

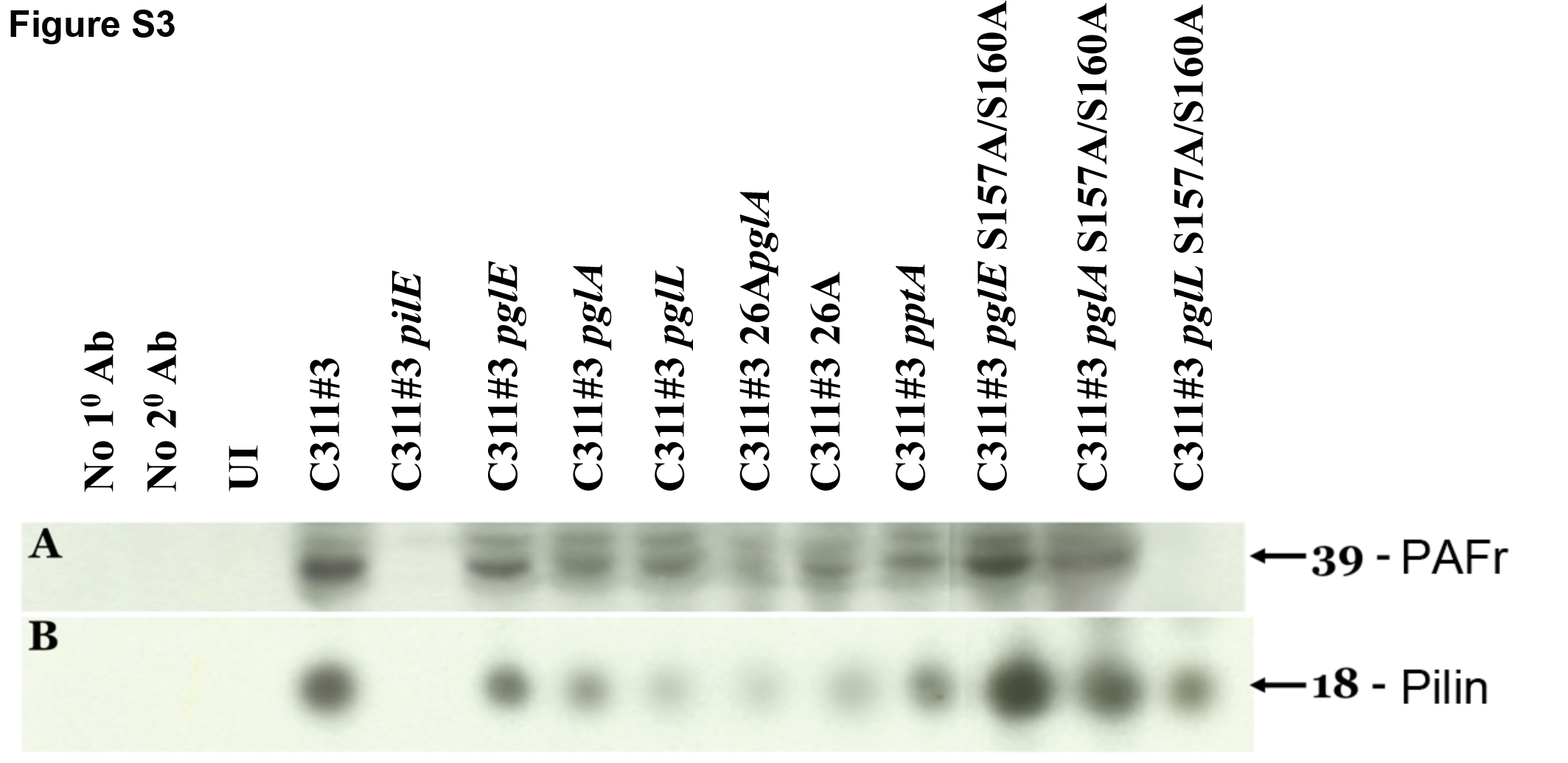

Supplement: Figure S3 — Immunoprecipitation of PAFr and pili from various C311#3 mutants and variants. Immunoprecipitation followed by Western Blot analysis demonstrates that meningococci bind to the PAFr via an interaction involving both the pili-linked ChoP and glycan modifications. Following a 15 minute challenge of 16HBE14 human bronchial epithelial cells, captured anti–pilin or –PAFr immunoprecipitates were transferred to a solid support medium and then subjected to western blotting with (A) anti-PAFr and (B) anti-pilin antibodies, as outlined in the text. The image shown was obtained from a single blot resulting from each immunoprecipitation condition; however, the centre portion of each blot (not relevant to this study) was deleted. No 1°Ab - the primary antibody was omitted from the initial immunoprecipitation capture step; No 2° Ab - the secondary, agarose-conjugated antibody was omitted from the immunoprecipitation assay; UI – uninfected cells. A panel of N. meningitidis C311#3 strains were evaluated for their ability to adhere to 16HBE14 cells via a pills-mediated mechanism and are indicated across the top panel. Pilin PTM for each strain are as follows: C311#3 WT (trisaccharide; ChoP+), C311#3pilE (pilin−; glycan−; ChoP−), C311#3pglE (disaccharide; ChoP+), C311#3pglA (monosaccharide; ChoP+), C311#3pglL(glycan−; ChoP+), C311#3 26ApglA (monosaccharide; ChoP−), C311#3 26A (trisaccharide; ChoP−), C311#3pptA (trisaccharide; ChoP−), C311#3pglES157A/S160A (disaccharide; ChoP−), C311#3pglAS157A/S160A(monosaccharide; ChoP−), C311#3pglLS157A/160A (glycan−; ChoP−). (TIF) [file ppat.1003377.s003.tif]
