# Supplementary material for: Dual Pili Post-translational Modifications Synergize to Mediate Meningococcal Adherence to Platelet Activating Factor Receptor on Human Airway Cells
Source: PLoS Pathog. 2013 May 16;9(5):e1003377. doi: 10.1371/journal.ppat.1003377 (PMC3656113; doi:10.1371/journal.ppat.1003377)
Supplement: Table S1 — Bacteria strains used. (DOCX) [file ppat.1003377.s006.docx]

**Table S1 Bacteria strains used**

| **Strain** | **Description** | **Source/Reference** |
| --- | --- | --- |
| ***Neisseria Meningitidis*** | | |
| C311#3 | Serogroup B strain with defined pilin-linked trisaccharide and phosphorylcholine. | (Virji, 1993) |
| C311#3Δ*pilE* | The gene *pilE* has been deleted by insertion of Km^R^ cassette | (Virji, 1993) |
| C311#3*pglA* | The gene *pglA* inactivated by insertion on Km^R^ cassette. It has pilin-linked monosaccharide, Km^R^ cassette | (Jennings, 1998) |
| C311#3*pglE* | The gene *pglE* inactivated by insertion on Km^R^ cassette. It has pilin-linked disaccharide, Km^R^ cassette | (Power, 2003) |
| C311#3*pglL* | The gene *pglL* inactivated by insertion on Km^R^ cassette. It has no pilin-linked glycan, Km^R^ cassette | (Power, 2006) |
| C311#3*pptA* | NMB0415 (*pptA*) inactivated by insertion on Km^R^ cassette. It has no pilin-linked phosphorylcholine. | (Warren, 2003) |
| C311#3*pptA::tet* | NMB0415 (*pptA*) inactivated by insertion on TetM cassette. It has no pilin-linked phosphorylcholine. | This study |
| C311#3 26A | NMB0415 (*pptA*) phase variation OFF | (Warren, 2003) |
| C311#3 26A*pglA* | NMB0415 (*pptA*) phase variation OFF. The gene *pglA* inactivated by insertion on Km^R^ cassette. It has pilin-linked monosaccharide, Km^R^ cassette | This study |
| C311#3*pilEHis* | The gene *pilE* has been tagged with Hisx6-RGS in the end of the gene. TetM as marker. | (Dieckelmann, 2003) |
| C311#3*PilETryp* | The gene *pilE* has inserted an extra tryptic cleavage site at Lys^56^. TetM as marker. | This study |
| C311#3*pilETrypFLAG* | The gene *pilE* has inserted an extra tryptic cleavage site at Lys^56^ and tagged with FLAG-tag (DYKDDDDK). TetM as marker. | This study |
| C311#3*pilETrypFLAG pptA* | The gene *pilE* has an inserted extra tryptic cleavage site at Lys^56^ and is tagged with FLAG-tag. TetM as marker. It has no pilin-linked phosophocholine, Km^R^ cassette. | This study |
| C311#3GFP | Wild type C311#3 contains the green fluorescent protein-expressing plamid pCmGFP | This study |
| C311#3*pglA*GFP | The gene *pglA* inactivated by insertion on Km^R^ cassette. It has pilin-linked monosaccharide. Strain also contains the green fluorescent protein-expressing plamid pCmGFP | This study |
| C311#3S157A/S160A | The gene *pilE* has point mutation sites at Ser157 and Ser160 to Ala157 and Ala160 TetM as marker. | This study |
| C311#3*PglA* S157A/S160A | The gene *pilE* has point mutation sites at Ser157 and Ser160 to Ala157 and Ala160 TetM as marker. The gene *pglA* inactivated by insertion on Km^R^ cassette. It has pilin-linked monosaccharide | This study |
| C311#3*PglE* S157A/S160A | The gene *pilE* has point mutation sites at Ser157 and Ser160 to Ala157 and Ala160 TetM as marker. The gene *pglE* inactivated by insertion on Km^R^ cassette. It has pilin-linked disaccharide. | This study |
| C311#3*PglL* S157A/S160A | The gene *pilE* has point mutation sites at Ser157 and Ser160 to Ala157 and Ala160 TetM as marker. The gene *pglL* inactivated by insertion on Km^R^ cassette. It has no pilin-linked glycan. | This study |
| C311#3TEPC+1 | C311#3 natural variant with trisaccharide, 2 ChoP on pilin, hyper-reactive to TEPC-15 antibody. | This study |
| C311#3TEPC+2 | C311#3 natural variant with trisaccharide, 2 ChoP on pilin, hyper-reactive to TEPC-15 antibody. | This study |
| C311#3TEPC+3 | C311#3 strain has lost a repeat unit leading to a frame-shift mutation and inactive PglE. As phase variation of *pglE* to an “off” state results in the loss of the terminal galactose from the pilin-linked trisaccharide. | This study |
| C311#3*pptA*11G | C311#3 strain contains an 11G tract in *pptA* had no detectable expression of ChoP on its pili. | This study |
| 8013SB | Serogroup C strain with defined pilin-linked disaccharide. This strains contains an 11G tract in *pptA* and had no detectable expression of ChoP on its pili. | (Marceau M, 1998) |
| 8013SB*pptA*8G | 8013SB strain contains an 8G tract in *pptA* and had detectable expression of ChoP on its pili | This study |
| 8013SB*pglL* | The gene *pglL* inactivated by insertion on Km^R^ cassette. It has no pilin-linked glycan, Km^R^ cassette. | This study |
| 8013SB*porA* | The gene *porA* inactivated by insertion on TetM cassette. It has no pilin-linked glycan, TetM cassette. | This study |
| MPJ11 | Serogroup B clinical isolate strain from cerebral spinal fluid (CSF). | (Berrington, 2006)(Power, 2003) |
| MPJ11*pglL* | The gene *pglL* inactivated by insertion on Km^R^ cassette. It has no pilin-linked glycan, Km^R^ cassette. | This study |
| MPJ11*pptA* | The gene *pptA* inactivated by insertion on TetM cassette. It has no pilin-linked phosphorylcholine. | This study |
| MPJ11*pglL/pptA* | A double knockout mutant. It has no pilin-linked glycan or phosphorylcholine. | This study |
| MPJ24 | Serogroup B clinical isolate strain from blood. | (Berrington, 2006)(Power, 2003) |
| MPJ24*pglL* | The gene *pglL* inactivated by insertion on Km^R^ cassette. It has no pilin-linked glycan, Km^R^ cassette. | This study |
| MPJ24*pptA* | The gene *pptA* inactivated by insertion on TetM cassette. It has no pilin-linked phosphorylcholine. | This study |
| MPJ24*pglL/pptA* | A double knockout mutant. It has no pilin-linked glycan or phosphorylcholine. | This study |
| MPJ26 | Serogroup B clinical isolate strain from CSF. | (Berrington, 2006)(Power, 2003) |
| MPJ26*pglL* | The gene *pglL* inactivated by insertion on Km^R^ cassette. It has no pilin-linked glycan, Km^R^ cassette. | This study |
| MPJ26*pptA* | The gene *pptA* inactivated by insertion on TetM cassette. It has no pilin-linked phosphorylcholine. | This study |
| MPJ26*pglL/pptA* | A double knockout mutant. It has no pilin-linked glycan or phosphorylcholine. | This study |
| MPJ50 | Serogroup B clinical isolate strain from CSF. | (Berrington, 2006)(Power, 2003) |
| MPJ50*pglL* | The gene *pglL* inactivated by insertion on Km^R^ cassette. It has no pilin-linked glycan, Km^R^ cassette. | This study |
| MPJ50*pptA* | The gene *pptA* inactivated by insertion on TetM cassette. It has no pilin-linked phosphorylcholine. | This study |
| MPJ50*pglL/pptA* | A double knockout mutant. It has no pilin-linked glycan or phosphorylcholine. | This study |

NB - Kan^R^ – Kanamycine resistant, TetM – Tetracycline resistant
