# Supplementary material for: Dual Pili Post-translational Modifications Synergize to Mediate Meningococcal Adherence to Platelet Activating Factor Receptor on Human Airway Cells
Source: PLoS Pathog. 2013 May 16;9(5):e1003377. doi: 10.1371/journal.ppat.1003377 (PMC3656113; doi:10.1371/journal.ppat.1003377)
Supplement: Table S2 — List of primers. (DOCX) [file ppat.1003377.s007.docx]

Table S2 List of primers

| Primer name | Sequence (5-3’) |
| --- | --- |
| PglE1F | AAGTAATGAAAATGTCGAACTTA |
| PglE3R | CGGAAATTTTCTAAATCGGCA |
| PilE-HisB | TCACCCAAGCTTTTAGCTATCACTTGCGT |
| PilE-HisC | ggatccatgaacacccttCAAAAAGG |
| PilE-TrypF | CACGGCAAATGGCCCGGGAAC |
| PilE-TrypR | GTTCCCGGGCCATTTGCCGTG |
| RfpB 14 | TTCGGAAACATTGTTCACAA |
| RfpB 9 | TTTTCGGCACTTTTGCCGCA |
| MWK1 | AACATTATGAGCCAAGCCTTACCC |
| MWK3^b^ | GTATCGGCGGCGCAGCAGGGGCTTTTCTTCCTGC |
| Ser 34 ⇒ Ala For | GCACAAGTTGCCGAAGCCATTCTTTTGG |
| Ser 34 ⇒ Ala Rev | CCAAAAGAATGGCTTCGGCAACTTGTGC |
| Ser45 ⇒ Ala For | CCGAAGGTCAAAAAGCAGCCGTCACAGAG |
| Ser 45 ⇒ Ala Rev | CTCTGTGACGGCTGCTTTTTGACCTTCGG |
| Ser 68 ⇒ Ala For | GCGTGGCAGCTTCTTCAACAATCAAAGGC |
| Ser 68 ⇒ Ala Rev | GCCTTTGATTGTTGAAGAAGCTGCCACGC |
| Ser 69 ⇒ Ala For | GCGTGGCATCTGCTTCAACAATCAAAGGC |
| Ser 69 ⇒ Ala Rev | GCCTTTGATTGTTGAAGCAGATGCCACGC |
| Ser 70 ⇒ Ala For | GCGTGGCATCTTCTGCAACAATCAAAGGC |
| Ser 70 ⇒ Ala Rev | GCCTTTGATTGTTGCAGAAGATGCCACGC |
| Ser157 ⇒ Ala For | CGACGCAGGTGATGCCAGCAGAGGATCG |
| Ser157 ⇒ Ala Rev | CGATCCTCTGCTGGCATCACCTGCGTCG |
| Ser160 ⇒ Ala For | GATGCCGGCAGAGGATCGCATCACC |
| Ser160 ⇒ Ala Rev | GGTGATGCGATCCTCTGCCGGCATC |
| PilE-NotI | GACCTGCAGGCGGCCGCGAATTCACTA |
| Tet-HindIII | CTCCACTGTTATATAATAAGCTTTCTGTTAAGG |
| FLAG-XhoI | TTAAAACTCGAGCTTATCGTCGTCATCCTTGTAATCGCTAGCATCACTTGCGTCGCGGCAGG |
| S157/160A-XhoI | AAAACTCGAGCTTATTAGCTGGCATCACTTGCGTCGCGGCAGG |
| *pptA*_EagI For | CAATTAACGGCCGAATAAAAGGAAGCCGATATGAAACAATCCGCCCGAATA |
| *pptA*_NcoI Rev | TGGAATCCATGGTCATTTTTTAGACGTATTTTTAGTCG |
| C311*pptA*11GFor | GGGGGGTATTTGCTGACGTAAAAAAC |
| C311*pptA*11GRev | CCCCCGATACACAATATTTCCAAAC |
| 8013*pptA*8GFor | GGGGTATTTGCTGACGTAAAAAAC |
| 8013*pptA*8GRev | CCCCGATACACAATATCTCCAAAC |
