# Supplementary material for: Dual Pili Post-translational Modifications Synergize to Mediate Meningococcal Adherence to Platelet Activating Factor Receptor on Human Airway Cells
Source: PLoS Pathog. 2013 May 16;9(5):e1003377. doi: 10.1371/journal.ppat.1003377 (PMC3656113; doi:10.1371/journal.ppat.1003377)
Supplement: Table S3 — PptA polymeric G screening of Neisseria MLST typed and clinical isolate strains. (DOCX) [file ppat.1003377.s008.docx]

**Table S3 PptA polymeric G screening of *Neisseria* MLST typed and clinical isolate strains.**

| Strains | Pili +/- | ChoP +/- | pptA polyG |
| --- | --- | --- | --- |
| C311#3 | + | + | 8 |
| 8013SB | + | - | 11 |
| MC58 | + | - | 9 |
| 6/88 | + | + | 8 |
| 107 | + | + | 8 |
| 2970 | + | + | 8 |
| 400 | + | + | 8 |
| 50/94 | + | + | 8 |
| H41 | + | + | 8 |
| H38 | + | + | 8 |
| Pmc12 | + | - | 7 |
| Pmc3 | + | - | 7 |
| IE30 | + | + | 8 |
| BZ147 | + | + | 8 |
| BZ133 | + | - | 7 |
| IF26 | + | - | 10 |
| Pmc17 | + | - | 7 |
| NGPB24 | + | + | 8 |
| B6/H6/77 | + | - | 11 |
| 1H15 | + | - | 9 |
| MPJ11 | + | + | 8 |
| MPJ1 | + | + | 8 |
| MPJ7 | + | + | 8 |
| MPJ13 | + | - | 7 |
| MPJ24 | + | + | 8 |
| MPJ26 | + | + | 8 |
| MPJ28 | + | - | 7 |
| MPJ30 | + | + | 8 |
| MPJ20 | + | - | 11 |
| MPJ62 | + | - | 9 |
| MPJ50 | + | + | 8 |
| MPJ47 | + | + | 8 |
| MPJ45 | + | + | 8 |
